# Spectral tuning and signaling of diverse and far red–sensitive rhodopsins in mantis shrimp eyes

**DOI:** 10.1126/sciadv.aef5666

**Published:** 2026-10-14

**Authors:** Camille Brouillon, Alina Pushkarev, Marjorie A. Liénard, Songhwan Hwang, Arno Munhoven, Richard McDowell, Robert J. Lucas, Megan Porter, Han Sun, Peter Hegemann

**Affiliations:** ^1^ Institut für Biologie, Experimentelle Biophysik, Humboldt-Universität zu Berlin, 10115 Berlin, Germany.; ^2^ GIGA Institute, University of Liège, 4000 Liège, Belgium.; ^3^ Leibniz-Forschungsinstitut für Molekulare Pharmakologie, Berlin 13125, Germany.; ^4^ Centre for Biological Timing, Department of Neuroscience, University of Manchester, Manchester M13 9PT, UK.; ^5^ School of Life Sciences, University of Hawai’i at Mānoa, Honolulu, HI 96822, USA.; ^6^ Institute of Chemistry, Technische Universität Berlin, Berlin 10623, Germany.

## Abstract

Stomatopod crustaceans (mantis shrimps) have extraordinary eyes with unmatched visual rhodopsin diversity, but their potential for broad color detection remains unclear. Here, we report that 17 rhodopsins, mostly from the midband eye region, are involved in color sensing and functionally diverse signaling via Gq/Gi coupling and describe the underlying mechanisms of spectral tuning. Two mid-wavelength rhodopsins preferentially absorb blue light and a majority of long-wavelength opsins absorb in the green range, but three absorb light beyond 600 nanometers, representing exquisite far red–sensitive animal rhodopsins. Several blue and red rhodopsins exhibit photoreversibility with opposite spectral shifts explained by retinal binding interactions, revealing unrecognized photochemical flexibility in stomatopod photoreception. Row-specific spectral sensitivity results from combining carotenoid filters with multiple individually tuned rhodopsins, altogether extending broad vision to the far-red spectrum.

## INTRODUCTION

Mantis shrimps are stomatopod crustaceans that have one of the most unusual and sophisticated visual systems among all known animals. The Caribbean species *Neogonodactylus oerstedii* (Fig. 1A) inhabits shallow-depth marine waters bathed with a broad spectrum of light, and its prominent highly mobile eyes bear at least 12 photoreceptor classes each sensitive to specific wavelength ranges between 300 and 700 nm, a remarkably high number unmatched thus far in animals, and whose role in color vision has long intrigued the scientific world (*1*). Photoreceptor diversity arises through three distinct regions, the dorsal and ventral hemispheres devoted to spatial and motion vision, and six central rows of ommatidia (the midband or MB) that run along the equator of the eye (Fig. 1B), with rows 1 to 4 mediating color discrimination (*2*), whereas rows 5 and 6 are responsible for polarization vision (*3*). In rows 1 to 4, three stacked tiers of cells act as successive filtering layers, each absorbing a portion of the light spectrum unavailable to the rhodopsin below (Fig. 1C). In rows 2 and 3, carotenoid-packed vesicles interspersed between the distal and proximal photoreceptive layers (*4*) considerably further tune the light available to their underlying photoreceptors.

**Fig. 1.**
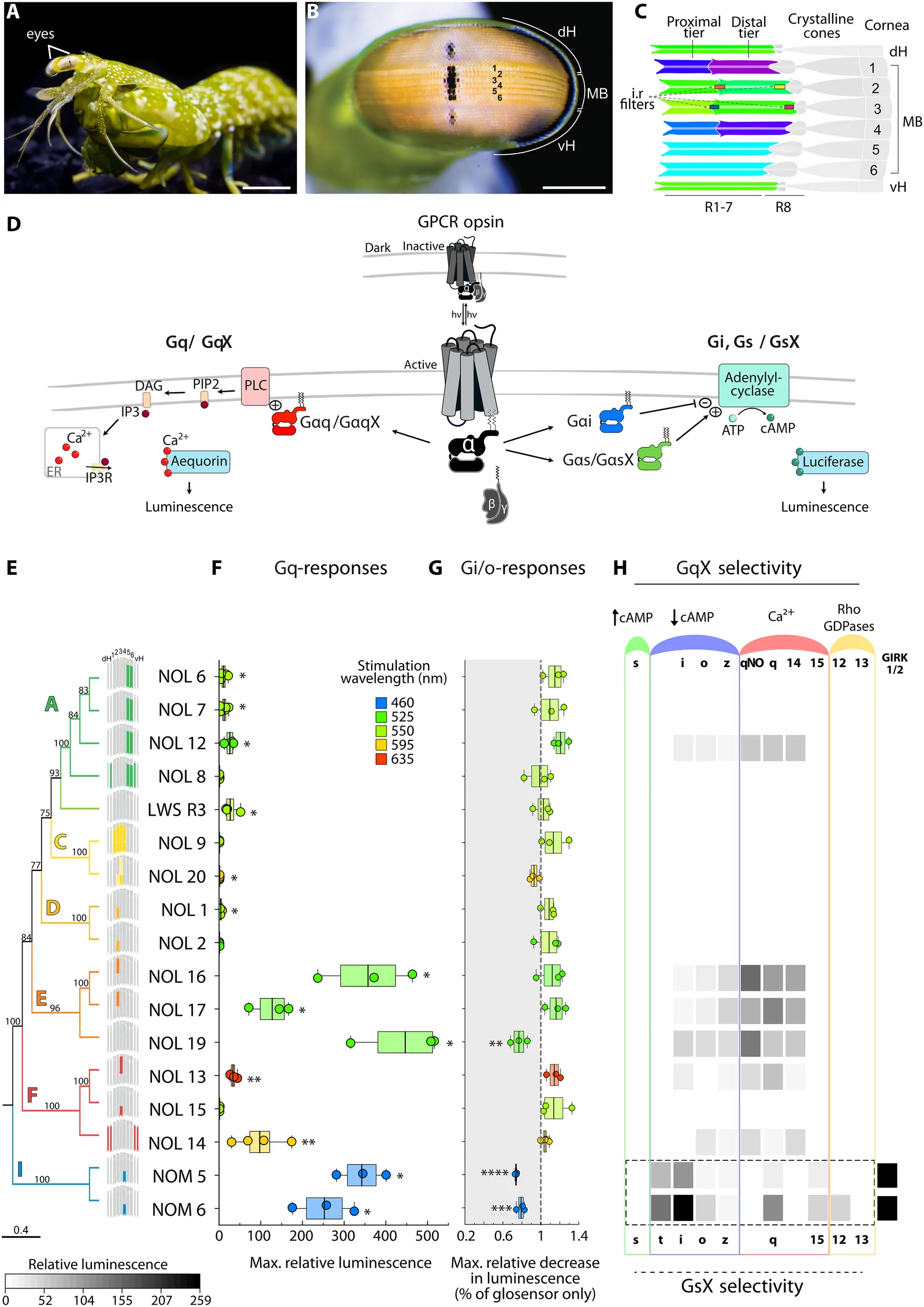
Mantis shrimp visual rhodopsins activate phototransduction through Gi and Gq signaling pathways. (**A**) Photograph of a *Neogonodactylus oerstedii* mantis shrimp (scale bar, 5 mm). Photo credit: Michael Bok, Lund University, included with permission. (**B**) Close view of the external division of the compound eye composed of 6 midband rows (MB), and dorsal (dH) and ventral hemispheres (vH) (scale bar, 1 mm) [taken from Porter *et al.* (*28*) with permission]. (**C**) Sagittal cross section diagram of the internal organization of the retina, including intrarhabdomal filters (i.r. filters), i.e., sitting within the rhabdom itself. Midband photoreceptive units are subdivided by tiers in rows 1 to 4 and are colorized to match maximal photoreceptor absorbance as measured by microspectrophotometry (*5*). (**D**) Scheme of bioluminescent second messenger assays with Gαq signaling via PLC and Gαs/Gαi stimulating or inhibiting adenylyl cyclase (see Materials and Methods for more details). (**E**) Localization patterns (scale bar, 200 μm) and evolutionary relationships from Porter *et al.* (*28*). (**F**) Summary of maximal relative aequorin Gq responses and (**G**) glosensor Gi/o responses (fig. S5), presented as mean ± SEM, compared to cells transfected only with luminescent reporters. Significant responses are marked with (*). (**H**) GqX- and GsX-protein coupling profiles of NOMs and NOLs.

In a seminal publication, Cronin and Marshall (*5*) measured bleaching spectra from *N. oerstedii* retinal ommatidial cross sections for all of the anatomically distinct photoreceptors absorbing within the visible spectrum. These spectra revealed absorbance maxima between 401 and 551 nm, presumed to correspond to one dominant rhodopsin (opsin with retinylidene chromophore) in each tier, under the assumption that all cells within a given tier expressed only a single one. The absorption spectra of the intrarhabdomal colored filter pigments were also measured to estimate the resulting sensitivities of midband receptors (*5*). These filters showed strong spectral overlap with their underlying photoreceptor sensitivity, an overlap that was expected to markedly reduce photon capture by up to three orders of magnitude (1000-fold) in the row 3 proximal tier (3P). This construction was proposed to confer red-light selectivity in 3P, but with very low overall sensitivity providing an adaptation potentially useful for vision under bright light conditions (*6*). Wavelength-dependent intracellular electrophysiological recordings (E-phys) of the ultraviolet (UV)–sensitive cells in *N. oerstedii* (*7*) and measurements spanning 400 and 700 nm for the underlying tiers in a number of related shallow- and deep-water stomatopods (*1*, *8*) support the idea that, together, the three tiers in each ommatidial row 1 to 4 of mantis shrimp eyes enable multichromatic sampling across the UV/Vis spectrum. Behavioral evidence further suggests spectral processing via color opponency and wavelength binning mechanisms (*9*, *10*).

Physiological evidence from several species further confirmed that mantis shrimp eyes can sense far-red light and led to the proposal that far-red sensitivity in row 3 results from strong intrarhabdomal filtering of a green-sensitive rhodopsin set as already known from other invertebrate eyes (*3*, *11*, *12*).

Most animal rhodopsins absorb maximally in the visible range (λ_max_ 400 to 580 nm), and many taxa also developed receptors sensitive to UV wavelengths (<400 nm) (*13*), but efficient red light–absorbing rhodopsins are remarkably rare in animals. So-called red-sensitive rhodopsins generally maximally absorb around 560 to 580 nm when using an 11-*cis* A1-retinal chromophore and only the far end of the rhodopsin absorbance spectrum reaches into the red part of the light spectrum (*14*–*19*). Extreme red sensitivity (>600 nm) is typically achieved either by coupling a long-wavelength sensitive (LWS) rhodopsin to a retinylidene chromophore with an extra double bond (11-*cis* A2-retinal) (*20*–*24*), by the addition of a bacteriochlorophyll light harvesting chromophore (*25*), or in combination with carotenoid oil droplet filters in birds, insects, and crustaceans (*6*, *26*, *27*).

A key missing piece of the puzzle is how these properties build upon the unique opsin diversity recently uncovered in the eye of *N. oerstedii*, with 33 RNA transcripts grouped into 17 distinct expression patterns and frequent tier coexpression across phylogenetic spectral clades (*28*). We sought to uncover how multiple specialized rhodopsins and the distribution of carotenoid pigments interact to tune color discrimination abilities in the mantis shrimp eye. Specifically, we set up to test whether functionally uncharacterized rhodopsins are efficient in detecting very long wavelengths of light to extend sensitivity into the far red.

## RESULTS AND DISCUSSION

### Three long-wavelength rhodopsins drive absorbance to the far red

To test the spectral coverage of the rhodopsins while eliminating the influence of filter components, we set out to fully characterize the remarkable rhodopsin diversity in vitro outside the tremendous complexity of the native eye. First, we compared the protein sequences (fig. S1) and phylogenetic relationships (Fig. 1E) of long-wavelength (NOLs) and middle-wavelength (NOMs) sensitive rhodopsin clades classified by the amino acid signature of the predicted retinal binding pocket. Specifically, in rhodopsins that absorb maximally between 440 and 600 nm, the connection of the deprotonated carboxylic acid (canonical counterion) to the protonated retinal Schiff base (RSBH^+^) and residues near the β-ionone ring are most critical for color tuning (*29*).

We selected and heterologously expressed the 17 opsins mostly found across the eye hemispheres and midband rows 1 to 4 used for color discrimination, comprising 15 full-length NOLs and the 2 NOMs of the row 4 proximal tier (fig. S1) (*28*, *30*). After reconstitution with the A1 11-*cis*-retinal used in crustaceans and most other invertebrates (*31*–*33*), the NOM5 and NOM6 opsins absorbed maximally at 455 and 459 nm in line with microspectrophotometric (MSP) data (*34*) and with their phylogenetic placement in middle-wavelength sensitive clades (Fig. 1E and fig. S2). Twelve NOL rhodopsins spanning stomatopod opsin phylogenetic clades A, C, D, E, and F (*28*) exhibited absorbance spectra with maxima near 540 nm (fig. S2). NOL14 (clade F) and NOL20 (clade C) formed LWS rhodopsins absorbing maximally near and above 600 nm (fig. S2). Similarly, although NOL13 (clade F) proved too unstable in solution to obtain a dark spectrum, action spectra measurements in human embryonic kidney (HEK) cells also consistently placed its λ_max_ at very long wavelengths (Fig. 2B). A plausible explanation for the instability of NOL13 in solution is its unusual high number of cysteine (Cys) residues (fig. S3), which might oxidize and destabilize protein structures in detergent (*35*). Despite these limitations, our combined absorption and action spectra measurements reliably show that these three rhodopsins are truly red sensitive (NOL13 λ_max_ = 615 nm, NOL14 λ_max_ = 592 nm, NOL20 λ_max_ = 607 nm) (Fig. 2B and fig. S2), whose contributions might have been previously overshadowed by abundant green rhodopsins in the eye or their exceptional instability in light-adapted animals (*34*). Notably, these red-shifted absorption maxima may even exceed the long-wavelength A3-type rhodopsins of *Apodemia* butterflies (λ_max_ = 600 to 605 nm) (*36*) and reach the purified L cone rhodopsins of salamander (615 nm) and goldfish (617 nm) both with A2-retinylidene chromophore (*26*).

**Fig. 2.**
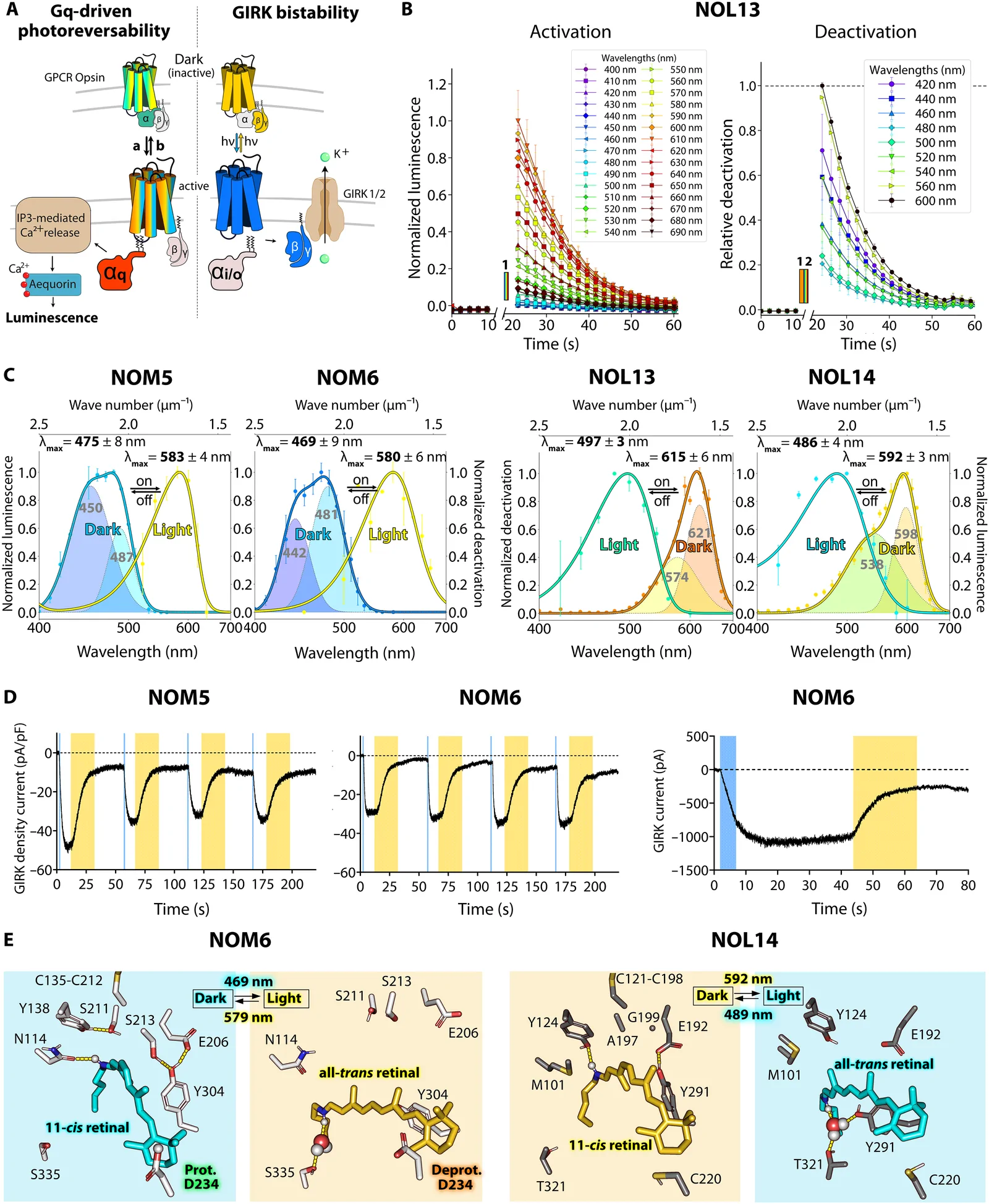
Photoreversible *N. oerstedii* rhodopsins have opposite spectral properties explained by distinct retinal Schiff base microenvironments. (**A**) Activation and deactivation of the Gq pathway (left) and the GIRK1/2 channel (right) by a Gq- and Gi/o-coupled GPCR opsin, respectively. (**B**) Wavelength-dependent aequorin luminescent activation/deactivation responses of NOL13. See the Supplementary Materials for other Gq-activated rhodopsins. Left: Activation is induced by a 1-s light pulse (colored rectangle 1) of fixed wavelengths (1 × 10^16^ photons cm^−2^ s^−1^, *n* = 3 experiments). Right: Deactivation is assayed by two consecutive 1-s light pulses, one with the fixed wavelength leading to maximal activation (orange rectangle 1, 600 nm, 1 × 10^16^ photons cm^−2^ s^−1^) followed by a second light pulse of varying wavelength (colored rectangle 2, 1 × 10^16^ photons cm^−2^ s^−1^, *n* = 3). (**C**) Activation and deactivation spectra of NOM5, NOM6, NOL13, and NOL14, derived from peak aequorin responses and fitted with a nonlinear least squares optimization of the Govardovskii visual template. Dark spectra are fitted with a two-component Gaussian Mixture Model (GMM) (table S2 and fig. S8). Data are presented as mean values ± SEM. The *x* axis is a linear energy scale (μm^−1^) corresponding to a log wavelength scale (nm). (**D**) GIRK photocurrents induced by the activation of NOM5 and NOM6 (1 s 470 nm light pulse, 1 × 10^14^ photons cm^−2^ s^−1^, blue line) and deactivated by a subsequent 20-s light pulse (595 nm, 4 × 10^15^ photons cm^−2^ s^−1^ yellow bar). (**E**) Predicted structural models of the inactive NOM6 and NOL14 with 11-*cis* retinal Schiff base (RSB) and active (all-*trans* RSB bound) states of NOM6 and NOL14. Key hydrogen-bonding interactions are shown as yellow dashed lines, and water molecules are represented as spheres (fig. S13).

### Long-wavelength rhodopsins couple mostly to Gq and middle-wavelength ones to Gi and Gq

We next wondered whether the *N. oerstedii* functional rhodopsins were actively involved in the phototransduction cascade. After cloning and construct optimization (fig. S4A), we tested G protein coupling and selective signaling preferences in each rhodopsin using bioluminescent second messenger assays in HEK293T cells (Fig. 1D). To first test individual rhodopsins coupling to each of the three main families of human G proteins, we tested the relative activation of Gq, Gi/o, and Gs (*37*) (Fig. 1, F to H, and fig. S5). Five NOL rhodopsins (2, 8, 9, 15, and 20) did not elicit measurable responses to any G protein type compared with cells transfected only with aequorin or glosensor, despite robust protein expression levels at the plasma membrane (fig. S6), suggesting that they do not couple to human G proteins. Ten NOL rhodopsins (1, 6, 7, 12, 13, 14, 16, 17, 19, and 20), LWSR3, as well as NOM5 and NOM6 coupled significantly to Gq (*P* < 0.04) (Fig. 1F, fig. S5, and table S1), although signal intensity between rhodopsins may not be directly comparable, notably owing to different expression levels, membrane targeting, and G protein coupling efficiencies. We could not detect activation of Gi or Gs for any NOL rhodopsin except for NOL19; however, NOM5 and NOM6 reacted readily with Gi (*P* < 0.001) (Fig. 1, F to H, fig. S5, and table S1).

We further investigated the G protein selectivity profiles by coexpressing *N. oerstedii* opsins in HEK293 cells that lack endogenous G proteins (*38*) but were complemented with individual human GsX chimeric proteins where the rhodopsin-interacting helix had been exchanged for helices of other G subtypes (*39*). Again, NOM5 and NOM6 both exhibited higher G protein coupling to Gsi than to Gst, whereas NOM5 had lower responses to GsX chimeras of the Gq family compared to NOM6 (Fig. 1H and fig. S5). As most of the signaling NOLs did not couple efficiently to GsX proteins but demonstrated substantial Gq responses, human GqX hybrids (*40*, *41*) were used following the same principle of the GsX assay (Fig. 1H). The Gq signal was quantifiable with enough variability to allow a reliable discrimination between GqX responses from six NOLs (NOL12, 13, 14, 16, 17, and 19) (Fig. 1H). All six characterized NOLs exhibited higher selectivity to GqX proteins of the Gq family, i.e., Gq or Gq14, but not Gq15 (Fig. 1H and fig. S5). In addition, all six NOLs showed weaker coupling to the Gi/o family of GqX proteins, with NOL14 in particular exhibiting comparatively high coupling to Gqo (21.9% of the sum of the GqX-protein selectivity responses) (fig. S5). None coupled significantly to GqX components of the G12/13 or Gs families. In particular, NOL13 activated preferentially Gq (43%) and G_qi_ (9.6%) but significantly less G_qz_ and Gqo (<8%) (Fig. 1F and fig. S5). We also tested the only so far reported endogenous *N. oerstedii* Gq protein (GqNO) putatively involved in phototransduction (*30*), which shares 83.9% amino acid identity with the mammalian Gαq protein used in our G-specificity assay including conserved interacting C-terminal residues (fig. S7). All six NOL Gq rhodopsins also activated mantis shrimp GqNO (Fig. 1H) but not with better signaling than human Gq, except for NOL16 and NOL19 (both 37% to GqNO and 17% to Gq) (fig. S5, D and E). Because of the strong Gq responses, we measured the wavelength dependence of Gq activation for NOL12, NOL16, NOL17, and NOL19, four green-sensitive rhodopsins expressed across rows 2, 5, and 6. We constructed individual action spectra that closely resembled the in vivo rhodopsin absorption profiles (*5*, *42*, *43*) (Fig. 2C and fig. S2), aligning both approaches to confirm these four rhodopsins’ green sensitivity. Overall, not including the fact that some unresponsive NOLs may couple to other potential native GqNO proteins, these results identify at least 13 functional rhodopsins actively coupling to G proteins, thereby parsimoniously contributing to phototransduction in the mantis shrimp eye.

### Four photoreversible rhodopsins of row 2 distal, row 4 proximal, and the periphery

Owing to its shallow water habitat and visual ecology, *N. oerstedii* is subject to fluctuating light and color distribution, which encouraged us to study the signaling specificity. To examine this question experimentally, we leveraged our Gq assay with the eight most promising, Gq-active rhodopsins (six NOLs and two NOMs), and implemented double pulse experiments to examine their photoreversibility properties, by delivering a first pulse of light for Gq activation, and a second pulse for deactivation, and by quantifying rhodopsin conversion back to their original D-state (Fig. 2, A and B) (*44*–*46*).

Wavelength-dependent activation and deactivation spectra were recorded for aequorin luminescent responses of NOM5, NOM6, NOL13, and NOL14 (Fig. 2C). We first activated NOM5 and NOM6 with a 1-s light pulse of fixed wavelength at near-saturating light intensity (450 nm) and tested their Gq responses after a second red-shifted 1-s light pulse of varying wavelengths. We found a maximal reduction of 28.1% of the initial response at 583 nm for NOM5 and 21.58% at 580 nm for NOM6 (Fig. 2C). Plotting the inverse of this reduction against wavelength reveals the active meta (L-) state spectra of the NOM photopigments that are photoreverted to the dark (D-) state with the second light pulse (Fig. 2C). The dark activation spectra presented a dual spectral composition that was best fitted with a two-component Gaussian model, suggesting a heterogeneity of the protein in the dark state (Fig. 2C, table S2, and fig. S8).

Similarly, NOL13 and NOL14 activation responses were acquired with a 1-s pulse fixed at 600 nm, and thereafter, their Gq-response reduction was quantified after blue-shifting the second light pulse, leading to maximal deactivation (75.1%) at 497 nm for NOL13, and reduced NOL14 response up to 55.7% at 486 nm. Visual fitting characteristics of the dark state again indicated the contribution of two spectral isoforms (Fig. 2C and fig. S8). In contrast, for NOL12, NOL16, NOL17, and NOL19, no second light pulse could change the response to the first, suggesting that either these rhodopsins are not photoreversible or have closely overlapping spectra in the dark and photo-activated states, or they thermally reconvert to the dark state in a few seconds.

Last, bistability and kinetic properties of NOM5 and NOM6 were further investigated electrically using the well-established G protein–gated inwardly rectifying potassium channel GIRK1/2, which canonically activates upon binding of the Gβγ subunit of Gi/o proteins (*47*) (Fig. 2B). The Gi-responsive NOM5 and NOM6 could repetitively activate and deactivate GIRK1/2, with blue and orange light, respectively, and with a limited loss in current amplitude, confirming that these rhodopsins are thermally photoreversible and bistable (Fig. 2D). Of note, even after using a 20-s pulse for deactivation at maximal light intensity (20 s, 4 × 10^15^ photons cm^−2^ s^−1^), GIRK photocurrents could never completely recover to the preillumination baseline, likely due to the still overlapping dark and photoproduct spectra at the absorbance tails and the high light intensity required to revert the rhodopsins to the dark state. Nevertheless, the photoproduct spectra of all four rhodopsins, which are considered as signaling states, were well separated from the dark state spectra (Fig. 2C). GIRK currents further indicated kinetics of light-induced activation-deactivation responses and the thermal stability of the signaling state for NOM5 and NOM6 (Fig. 2D). Thus, NOM5, NOM6, NOL13, and NOL14 are not only stable after photoexcitation without bleaching, but also capable of returning to their inactive dark state photochemically due to the weak overlap of the D- and L-spectra.

### Inverse spectral tuning of NOM6 and NOL14 arises from specific retinal binding pocket properties

To gain a better understanding of the pronounced spectral differences between the dark states (D) and photoproducts (L), as well as the inverted photochemical shifts observed between NOM6 and NOL14, we generated structural models of the retinal binding pockets while maintaining the retinylidene chromophore protonated (Fig. 2E and fig. S9). The structural transitions from 11-*cis* retinal in the D-state to all-*trans* retinal in the L-state were modeled by combining AlphaFold3 predictions with multiple sequence alignment (MSA) manipulation (*48*, *49*), molecular docking, all-atom molecular dynamics (MD), hybrid quantum mechanics/molecular mechanics (QM/MM) simulations, and vertical excitation energy calculations.

The predicted 7TM helix structure models exhibit high overall confidence, with predicted local distance difference test (pLDDT) values mostly above 80, and high structural stability during equilibrium MD simulations (figs. S9, S10, and S11). In the model, the canonical counterions E206 and E192 are positioned at distinct large distances from the retinal Schiff base in both proteins, which are 8.2 and 6.5 Å in the D-states of NOM6 and NOL14, and 14 and 8.1 Å in the corresponding L-states, respectively. These distances are too large for direct hydrogen bridges and need other residues or water in between; the influence of the charge is expected to be very small. For comparison, the experimentally determined D-state of the jumping spider rhodopsin (PDB entry 6I9K) exhibits a distance of 6.7 Å, and E194 is connected to the RSBH^+^ via a water and Ser^199^. Instead, NOM6 features a unique acidic residue (D234) near the β-ionone, which is replaced by a polar cysteine (C220) in NOL14. This location is one of the two that have been widely used by nature and bioengineers to fine-tune rhodopsin absorption. In most blue-sensitive rhodopsins, these sites are occupied by hydrophobic residues, whereas in red cone rhodopsins (including human), they are occupied by hydrophilic residues (*29*, *50*). A negatively charged residue at this position as found in NOM5/6 is however unique so far.

Comparison of the predicted absorption spectra for different protonation states of D234 in NOM6 revealed that a protonated D-state and a deprotonated L-state showed the closest agreement with the experimental data (fig. S12) in agreement with the original double-point charge model proposed for rhodopsins (*51*, *52*), in which a negative charge near the β-ionone electrostatically stabilizes the positive charge that formed in this region upon retinal photoexcitation causing a strong red shift. Next, we sought to rationalize the inverse spectral shifts observed between NOM6 and NOL14 by comparing their QM-optimized structural models. Notably, in NOM6, the residues flanking the disulfide bond C135-C212 are polar (S211 and S213) and S211 forms a hydrogen bond with Y138, while S213 interacts with Y304 and, indirectly, the counterion E206. The S211-Y138 interaction redirects the N-H of the retinal to N114 increasing polarity at the retinal nitrogen (fig. S13). In contrast, in NOL14, the disulfide neighbors are nonpolar (A197 and G199), and the 11-*cis* retinal N-H occasionally binds with Y124, while Y291 interacts with the counterion E192 only. The resulting reduced retinal distortion leads to greater π conjugation and a red shift, compared to the distorted C12-C15 bonds in NOM6 (fig. S14). The all-*trans* retinylidene N-H^+^ group in NOL14 forms a water-mediated hydrogen bond with T321, and occasionally to Y291. This configuration electrostatically stabilizes the ground state, increasing the excitation energy and causing a blue shift while the deprotonated D234 near the ring in NOM6 exerts the opposite effect, inducing a red shift. In conclusion, we propose that the inverse spectral shifts observed between NOM6 and NOL14 arise from substantial differences in the polarity and hydrogen-bonding networks within their retinal binding pockets. Such variations modulate the extent of π conjugation and geometric distortion of the retinal, thereby fine-tuning its absorption characteristics.

### Modeling the tuning of rhodopsin sensitivity with and without filtering pigments partitions photoreceptor spectral specialization

Next, we investigated how the multiple rhodopsins of the eye hemispheres and midband ommatidial tiers tune photosensitivity without or under the influence of inter-rhabdomal filtering pigments, respectively. In the lateral dorsal and ventral hemispheres (DH and VH) devoid of inter-rhabdomal filtering, NOL14 with λ_max_ = 592 nm is expressed in conjunction with NOL8 with λ_max_ = 540 nm. Together, they confer an unfiltered broad sensitivity (Fig. 3A), which is at least half-maximal between 470 and 660 nm. This offers the dorsal and ventral sides of the mantis shrimp eye an exceptional ability to perceive light of a broad spectrum ranging from blue to very long wavelengths, the latter possibly persisting at very shallow depths, especially at dusk and dawn (*11*, *53*), quite in line with the computed sensitivity function of the periphery (*5*) and the direct electrical recordings from *Gonodactylus chiragra* (*54*)*.* We next calculated the resulting spectral sensitivity of MB row 2 distal tier (2D), where NOL16, NOL17, and NOL9 (λ_max_: 521, 537, and 541 nm, respectively) are expressed in conjunction with F1 filters (R2F1) (Figs. 3 and 4A). NOL9 is present in all midband rows 1 to 4 (*28*) and presumably less likely to contribute to major differences in tier photoreceptor sensitivity. By taking into account the reported absorption spectra of R2F1 (*5*), our results provide evidence that the maximal sensitivities of NOL16 and NOL17 are reduced by the R2F1 filter to roughly 45% and shifted bathochromically by 16 to 18 nm (Fig. 4, B and C, and fig. S15). In addition, R2F1 narrows half-bandwidth (HBW) from 100 nm to 60 to 80 nm, substantially increasing spectral discrimination in row 2 without great sensitivity loss (fig. S15).

**Fig. 3.**
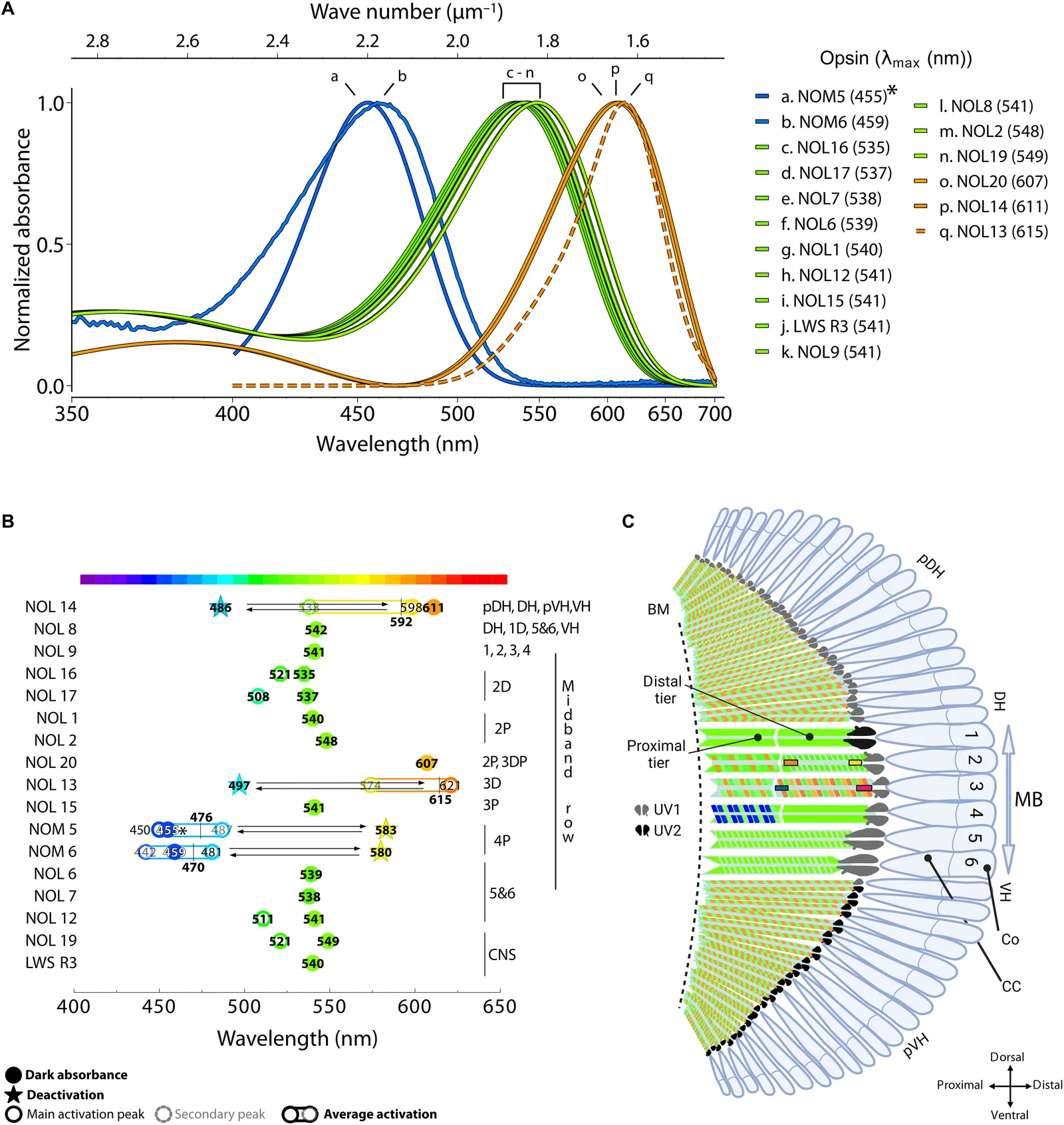
Multiple specialized spectral and photoreversible rhodopsins contribute to sharp, finely tuned photoreceptor properties in the mantis shrimp retina. (**A**) Spectral fits dark NOL and NOMs absorption with 11-*cis* retinal or 9-*cis* retinal (*) full lines. Dark spectra before fit are presented in the Supplementary Materials (fig. S2)*.* For NOL13, the action spectrum (Fig. 2C) is plotted in a dashed line instead of the dark spectrum due to the rhodopsin’s instability in detergent. (**B**) Absorbance maxima of mid- and long-wavelength rhodopsins characterized in this study, arranged by localization in different regions including eye hemispheres, eye midband rows 1 to 6, or the central nervous system (CNS). DH, dorsal hemisphere; VH, ventral hemisphere; p, peripheral; D, distal tier; P, proximal tier. Numbers indicate best-fit estimates of λ_max_ from dark absorbance (full circles), plus activation (empty circles), and deactivation spectra (stars) when measurable. Maximal activation for NOL13, NOL14, NOM5, and NOM6 is reported as the peak of the Gaussian mixture (main individual peak in empty circle and secondary individual peak in light gray and dashed circles) in bold, circled by an ellipse (see table S2). Bidirectional arrows indicate that NOM5, NOM6, NOL13, and NOL14 can be reversibly switched bidirectionally between dark and active states. (**C**) Sagittal cross section diagram of the eye. The location and representative absorption range of the multiple rhodopsins expressed in each region are mapped as colored diagonal cross patterns according to the number of rhodopsins expressed in this study and their absorption. Intrarhabdomal filters in rows 2 and 3 of the midband row (MB) are depicted by vertical rectangles. Created in BioRender. Pushkarev, A. (2026) https://BioRender.com/rolv4o0. Co, cornea; CC, crystalline cone; BM, basal membrane.

**Fig. 4.**
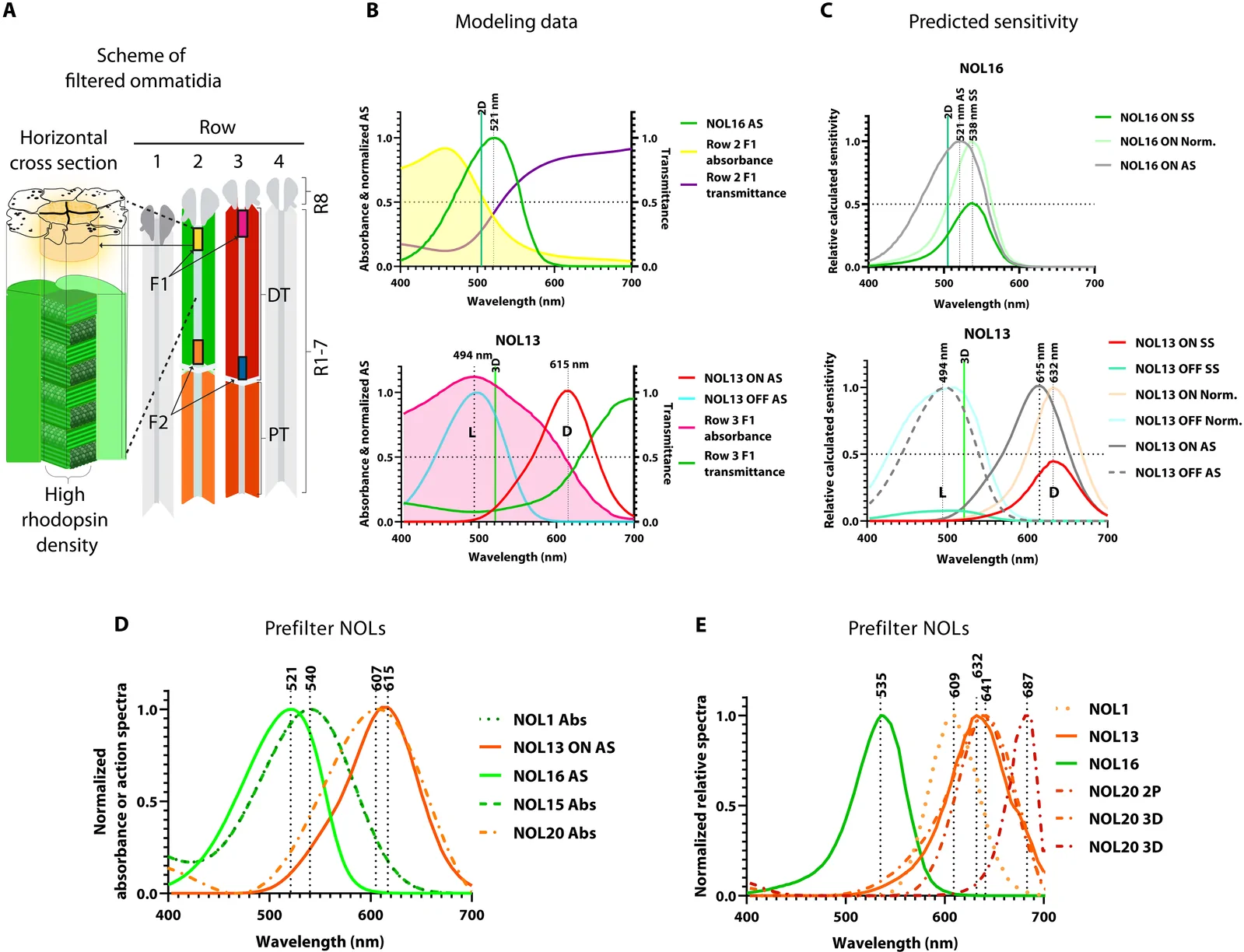
Long-wavelength rhodopsins of midband rows 2 and 3 are fine-tuned by intrarhabdomal filters, extending sensitivity to the far red. (**A**) Scheme of midband rows 2 and 3 showing the intrarhabdomal carotenoid filters (F1 and F2) and the microvillar organization. DT, distal tier; PT, proximal tier. Tiers in rows 2 and 3 are colored according to the spectral sensitivity obtained with quantitative modeling of rhodopsin filtering. Created in BioRender. Pushkarev, A. (2026) https://BioRender.com/sjqhk9i. (**B**) Filter absorbance and transmittance (*5*), and wavelength absorbance of NOL16 (top) and NOL13 (bottom) of rows 2 and 3 are plotted against wavelength. MSP peak absorbance of the distal tiers indicated by the vertical line in turquoise (2D) and green (3D) (*5*). L, light (ON); D, dark (OFF); AS, action spectra. (**C**) Calculated relative spectral sensitivity for NOL16 (top) and NOL13 (bottom) resulting from transmittance of the F1 filter multiplied by normalized action spectra of NOL16 and NOL13, respectively. Faint lines show the normalized sensitivity. For NOL13, activation and deactivation spectra are plotted in red and blue, respectively, and the action spectra are plotted in gray. Photoreceptor sensitivities and filtering in the UV are neglected. Half-bandwidth is indicated with a bold horizontal dashed line. Norm., normalized; SS, spectral sensitivity. Sensitivities of individual additional rhodopsins are presented in the Supplementary Materials (fig. S15). (**D** and **E**) Normalized spectral sensitivities of tiers 2D, 2P, 3D, and 3P. (D) Original rhodopsin spectra before i.r. filtering. (E) Spectral sensitivity postfiltering. Maximal peak sensitivities are marked by dotted vertical lines. Postfiltering sensitivity below 0.1% was not plotted (e.g., NOL15). AS, action spectra; Abs, absorbance spectrum.

In MB tier 3D, comprising NOL9, NOL20, and NOL13, the F1 filter (R3F1) absorbs further to the red end of the visible spectrum compared to R2F1 (Fig. 4B and fig. S15). Therefore, in the dark, the spectral sensitivity of NOL13 and NOL20, both red shifted in absorption compared to NOL16, are still screened out by R2F1. Likewise, the resulting NOL13 and NOL20 rhodopsin sensitivities in 3D are shifted bathochromically by 16 nm, respectively, achieving 632 nm for NOL13 and 637 nm for NOL20, on the expense of a final moderate reduction of sensitivity to 45 to 49% (Fig. 4, C and E, and fig. S15D). Because the absorption maxima of NOL16 and NOL13 are at a wavelength where transmission of the F1 filters changes the most (fig. S15D), it causes compression of the HBW and shifts the sensitivity peaks of the rhodopsins with only moderate sensitivity loss (fig. S15, C and D).

Earlier microspectrometric data analysis in *N. oerstedii* predicted 560 nm as peak sensitivity for 3D (*6*), whereas electrical spectral sensitivity measurements of two related stomatopods, *Gonodactylus smithii* and *Haptosquilla trispinosa*, placed λ_max_ at 610 nm for row 3D (*8*). Our calculations show that modulation of photoreceptor red light sensitivity in 3D falls above the previously proposed amplitude range for peak sensitivity with 632 nm for NOL13 and 637 nm for NOL20 in 3D (Fig. 4), demonstrating the dual role of R3F1 filtering and the multiple rhodopsin sensitivities. Reversely, we find that the light sensitivity of the NOL13 photoproduct (NOL13-L, λ_max_ = 497 nm) is more strongly reduced to 7.6% at its maximum, proportional to the low R3F1 filter transmission at these wavelengths (Fig. 4, B and C).

In the MB proximal tiers in row 2 (2P) and row 3 (3P), sensitivity calculations account for the reduction in light spectrum available after absorption in the distal tiers and the high optical densities of the proximal filters [*A* = 7.7 for R2F2 and *A* = 3.6 for R3F2 (*5*)] that further markedly reduce the light captured by the stacked proximal tier rhodopsins. In doing so, we show that in 2P, R2F2 filtering of NOL1 (λ_max_ = 540 nm) and NOL20 (λ_max_ = 607 nm) reduces their maximal sensitivities to 2.8 and 20%, respectively, and shifts the narrowed maxima to 609 and 641 nm (fig. S15D). In 3P, R3F2 filtering of NOL15 shields out the rhodopsin sensitivity even further to <0.1% due to strongly overlapping absorbance maxima between the rhodopsin and the filter, whereas filtering of NOL20 shifts the tier sensitivity maxima to 687 nm with a reduction to ∼2.5% (fig. S15). This interplay acts as the mechanism to achieve in vivo far-red sensitivity in row 3 in the *N. oerstedii* eye*.*

### Trade-off photoreceptor adaptations between spectral reach and sensitivity allow vision at the far-red edge

Our findings show that the mantis shrimp *N. oerstedii* uses a subset of long-wavelength rhodopsins (NOL13, NOL14, and NOL20) that absorb light maximally near or above 600 nm (Fig. 3A and fig. S15), achieving real far-red absorbing properties by sequence modification while still coupling to the universal A1 11-*cis*-retinal chromophore. In the midband rows 2 and 3 specialized to color vision, light at the red edge of the rhodopsin absorption spectrum can be effectively used by the employment of selective filters that absorb shorter wavelengths of light through red carotenoid pigments (Fig. 3 and fig. S15) (*5*, *36*, *55*, *56*). This mechanism confers *N. oerstedii* with exceptional long-wavelength (far red) vision, but reduces absolute sensitivity. Hence, the magnitude of the resulting shift in effective spectral sensitivity depends on the degree of overlap between the filter’s absorbance spectrum, the rhodopsin absorbance, and on the filter’s optical density. High optical density and asymmetric filter-rhodopsin absorbance overlap produce large shifts (e.g., NOL1, NOL13, and NOL20), whereas well-aligned rhodopsin-filter absorption will cause a loss in photon catch and attenuate light sensitivity of the corresponding tier with little or no shift (e.g., NOL15) (Fig. 4 and fig. S15). In the proximal (P) tiers of color-vision rows 2 and 3 (2P and 3P), the intrarhabdomal filters are particularly optically dense, leading to substantial spectral shift reaching up to 80 nm but sacrificing sensitivity down to <0.1%. Consequently, far-red vision is likely restricted to bright daylight, when using 2P and 3P photoreceptors, after the distal 2D and 3D rhodopsins are already inactivated in their L-state and allowing light transmission to the P-tiers. In contrast, at lower light levels, during dusk and dawn, *N. oerstedii* is reported to be especially active and ventures further from its burrow before sealing itself for the night (crepuscular activity) (*57*). Color vision thus relies mainly on 2D and 3D that provide a less extreme, but a more sensitive spectral range, allowing *N. oerstedii* to take advantage of times of day when it is safer from predators and food is more available.

Overall, in *N. oerstedii*, the distal tiers in rows 2 and 3 of the midband ommatidia with high sensitivity are designed for low light intensities, whereas the proximal tiers are the main actors at bright light with narrowed but strongly reduced light sensitivity and a far-red maximum near 690 nm.

Mantis shrimps can therefore sample far-red wavelengths more specifically than most other animals and have the visual capacity to potentially exploit far-red chromatic and achromatic signal reflectance (total luminance), although its contribution in an ecological context is untested behaviorally. For intraspecific communication, stomatopods reliably assess total changes in light reflectance in raptorial appendage regions of competitors including meral spots (*58*, *59*), which, in *N. oerstedii*, contribute UV reflectance, and up to 10% far-red light between 700 and 800 nm (*59*, *60*). This suggests that its eye far-red sensing photoreceptors may add a signaling channel in specific ecological and visual contexts, alone or combined with UV cues or fluorescent enhancement, or at high sun angles when UV and visible spectral channels are relatively bleached and filtered out.

### Spectrally separated rhodopsin states in the visible spectrum likely contribute to color recognition

The photoreversibility properties of at least four mantis shrimp rhodopsins are unique to date in the sense that they are not only bistable, but they are also bidirectional photoswitchable with the spectra of dark and signaling states well separated within the visible range. This separation sets them apart from many bistable invertebrate rhodopsins like parapinopsins (*61*) or *Platynereis* rhodopsins (PdCO) (*62*), where one state absorbs in the visible and the other in the UV, and squid and jumping spider rhodopsins (*63*), where both D-state and L-state absorb in the visible with strongly overlapping spectra (*45*, *64*, *65*).

In mantis shrimp, photoreversibility with large spectral separation of the D- and L-states realized without chromophore deprotonation seems to be achieved by a unique hydrogen-bonding network that fine-tunes the electronic environment and geometric distortion of the chromophore, along with large modulation upon photoisomerization, but without large alteration of the retinal Schiff-base p*K*_a_. Although a small part of the spectral shift may arise from differences in retinal-counterion distance, the pronounced spectral distinction between the D- and L-states is an unprecedented rearrangement of the hydrogen-bonding network that represents an ingenious evolutionary solution for bidirectional photoswitching and to open in the eye spectral windows for the more distant rhodopsins underneath. In rhodopsins of jumping spider or octopus, the only small shift prevents photoreversibility almost completely (*63*, *66*). Collectively, the results of our current study show that the finely tuned visual system of the mantis shrimp *N. oerstedii* achieves exceptional color discrimination notably via a diverse functional repertoire of middle- and long-wavelength rhodopsins, some of which evolved photoreversibility and far-red absorption properties. Photoreversible middle- and long-wavelength rhodopsin states are expected to contribute to rapid contrast perception and spectral tuning, whereas far-red rhodopsins act in concert with carotenoid filtering to approach maximal sensitivity near 690 nm. Low-density filters of the distal areas shift the spectra and reduce the sensitivity less and should function in dim light whereas the high-density filters of the proximal tiers shift more by sacrificing sensitivity, which is affordable in bright light, illustrating a series of evolutionary innovations distinct from typical animal rhodopsins, and that may carry highly beneficial adaptations in bright, spectrally broad and complex underwater environments.

## MATERIALS AND METHODS

### Experimental design

The purpose of this study was to characterize the function of multiple rhodopsins primarily expressed in the color-vision rows, i.e., the central midband of the eye of the stomatopod crustacean *N. oerstedii*, and to explain the mechanistic interplay between rhodopsins and intrarhabdomal filtering pigments in achieving both broad spectral sensitivity and very long–wavelength sensitivity, which together link the molecular and physiological mechanisms underlying its remarkable color vision. Heterologous expression was used to determine the absorbance and G protein coupling of individual rhodopsins outside of the complex eye environment. Bioluminescent in vitro assays were used to identify signaling rhodopsins and evaluate G specificity using native and chimeric G proteins. Electrophysiological recording was used to identify well-separated activation and deactivation spectra of a subset of photoreversible rhodopsins with inverse spectral properties, and thereafter electrical voltage clamp experiments allowed us to study rhodopsin kinetics of GIRK-channel activation. Structural and molecular modeling informs on the inner workings likely controlling inverse spectral properties. Last, rhodopsin sensitivity was modeled with and without filtering pigments, informing on photoreceptor specializations to a variable light spectrum to provide a functional and ecological context.

### Phylogenetic analyses

*N. oerstedii* opsin DNA sequences were obtained from NCBI and reanalyzed in Geneious Prime (Version 2026.1.2) to exclude partial sequences (data S1). Full-length opsins were selected if they met further criteria: belonging to the proposed long-wavelength (NOL) or middle-wavelength (NOM) clades and being expressed either in the color absorbing midband rows, the lateral hemisphere of the mantis shrimp eye (NOL14), as well as in the eye stalk (LWS R3). Deduced protein sequences for 17 selected opsins (14 NOLs, 2 NOMs, and LWS R3) were aligned in ClustalX (*67*), and the phylogeny was reconstructed as reported earlier (*28*).

### Opsin cloning and expression constructs

Opsin ORF sequences from *N. oerstedii* were codon optimized for mammalian expression, synthesized by Genscript and subcloned into various expression vector constructs (fig. S2A), using PCR amplification followed by restriction digestion and T4 ligation or Gibson assembly (*68*). The coding sequence for the Gαq protein from *N. oerstedii* was codon optimized and subcloned into the pCAGGS plasmid vector. GqX plasmids were obtained from Asuka Inoue (Tohoku University, Japan). All final plasmids were sequence verified by Sanger sequencing, and endotoxin-free plasmid stocks were prepared using the Zymo Midi-prep procedure (Zymo Research) before transfection.

### Cell culture

HEK293T cells (Life Technologies) were used for opsin purification; Gq, Gi/o, and Gs live bioluminescent second messenger assays and HEK293 lacking GNAS/GNAL/GNAQ/GNA11/GNA12/GNA13/GNAZ (HEK293ΔG7) (*69*) were used for testing more specifically the Gα signaling selectivity in GsX and GqX assays. Cell culturing and expression procedures for rhodopsin purification and dark absorbance measurements are described in Supplementary Materials and Methods.

### Bioluminescent plate reader experiments

Luminescence was measured from a single well in a plate reader (Infinite 200Pro, Tecan) with an integration time of 1 s every 0.5 s for Gq assays and every 60 s for Gs and with an integration time of 300 ms every 0.5 s for Gi assays. Dark luminescent baseline was measured for five cycles until the measurement was paused and the plate was ejected for illumination.

For Gq assays, one well was subjected to a 1-s illumination using a pE-4000 (coolLED Ltd.) and measured. For Gs assays, luminescence was measured for all wells before ejection. One-second light pulse was applied subsequently to each well before the measurement was resumed.

For Gi assays, 2 μM forskolin (Sigma-Aldrich) was added to each well to elevate cAMP levels and recording was resumed for 20 to 30 min and illuminated as before. In the three assays, luminescent responses were compared to cells transfected only with the corresponding luminescent reporter, subjected to the same illumination conditions as the opsin.

### Bioluminescent GsX and GqX assays

Luminescence was measured for 1 s every 60 s. Preillumination baseline was recorded for five cycles before recording was paused and the plate was ejected. Light-dependent GsX responses were acquired using light-titration curves. Each well was exposed once to a single light intensity pulse of the same wavelength, starting from the highest intensity and gradually decreasing in 0.5 log_10_ steps to capture the peak response, especially small signals from low intensities.

As NOL opsins did not signal efficiently through GsX proteins, GqX chimeric proteins were used instead, following the same principle. Plasmid DNAs encoding Gαq proteins with the last five amino acids exchanged with the ones from other Gα subfamilies (gift from A. Inoue, Tohoku University, Japan), as well as a native *N. oerstedii* G protein alpha subunit (GqNO, Genbank Acc. no. AUC64085.1) were cotransfected individually with a given rhodopsin, and transfected cells were resuspended in 96-well plates as described previously (*41*). Light-dependent GqX responses were acquired using light-titration curves. Similarly to the Gq assay, each well was exposed once to a 1-s light pulse of a fixed wavelength and measured at a time with different light intensities, starting from the lowest and increasing in 0.5 log_10_ steps to avoid cross-light contamination of the neighboring wells.

### Action spectra measurements

Action spectra were measured using the Aequorin Gq-assay. Light stimulations were delivered using a Polychrome V (TILL Photonics) in combination with neutral density filters to keep an equal photon flux across wavelengths. A 3D-printed holder was built and fixed at the end of the light guide to hold ND filters of varying transmittance (Newport) and maintain a fixed distance between the light source and the sample. A controllable motorized slit was incorporated into the holder to allow precise timing of stimulation and to prevent unwanted stimulation at the resting wavelength of the Polychrome.

To measure activation spectra, a 1-s light pulse of a fixed wavelength was delivered to one well from 400 to 690 nm in 10 nm steps. Each well was illuminated and measured once before changing the wavelength and continuing to the next well. The activation spectra were reconstituted using the maximal relative increase in luminescence for each wavelength, normalized between the minimal and maximal peak response. Data points were fitted with a nonlinear least squares optimization of the Govardovskii visual template (*70*) in python 3.12 using SciPy 1.14 (*71*) and scikit-learn (*72*) with initial parameter guesses based on the experimental peak wavelength. For NOM5, NOM6, NOL14, and NOL13, dark action spectra were fitted with a Gaussian Mixture Model with two components. The λ_max_ and parameters values, along with their uncertainties, were determined from the peak of the fitted curves using 1000 bootstrap resampling iterations, and the *R*^2^ score was calculated to assess the quality of the fit.

To measure deactivation spectra, wavelength bandwidths were set to 15 nm to ensure sufficient deactivation. A first 1-s nearly saturating light pulse was delivered to activate the opsin at a fixed wavelength close to the λ_max_, followed by a consecutive 1-s light pulse of 1 × 10^16^ photons cm^−2^ s^−1^ varying wavelength. The relative deactivation was calculated from the dual stimulation peak response expressed as a percentage of the single stimulation peak response and fitted similarly to the activation spectra.

It is important to note that the decay kinetics of the aequorin luminescent signal in the Gq-coupled assay does not directly correspond to opsin deactivation because the calcium levels are quickly down-regulated by cellular mechanisms, independently of the sustained activation of the opsin. In addition, the intracellular calcium response triggered by the first light pulse rises and falls too rapidly for the second pulse to precisely probe the opsin’s active state. This means that the reduction in luminescence response observed after the second pulse may reflect an interruption of the ongoing calcium signaling initiated by the first pulse, compounded by the regulation of calcium levels.

We also note that peak sensitivities extracted from action spectra did not strictly coincide with maxima measured from dark absorbances, which may be attributable to the surrounding lipid environment in the cell membrane, as in vivo opsin properties such as stability, function, and absorption may differ slightly in detergent-based buffer systems (*73*).

### GIRK whole-cell patch-clamp experiments

HEK293 cells stably expressing the GIRK1/2 channel (HEK293-GIRK1/2, gift from S. Herlitze, Ruhr-Universität Bochum) were used to measure NOM5- and NOM6-induced photocurrents. Cells were cultured in DMEM supplemented with Geneticin (G418, Brand) and seeded into 12-well plates at a density of 350,000 cells per well. Cells were transiently transfected individually with pIRES-CMV-Lucyrho-NoM5-TS-IRES-Turbo635 and pIRES-CMV-Lucyrho-NoM6-TS-IRES-Turbo635 construct B1 (fig. S4) using FuGENE HD (Promega). Cells were incubated in the dark for 3 to 7 hours and resuspended using Trypsin-EDTA. They were then transferred to 15-mm coverslips coated with poly-D-lysine (Sigma-Aldrich) in 35-mm petri dishes (TPP) and supplemented with 2 μM 11-*cis*-retinal under red dim light. Twenty-four hours after transfection, cells were recorded at room temperature in whole-cell patch-clamp experiments using patch pipettes of resistance between 2 and 5 MΩ filled with intracellular solution [140 mM KCl, 3 mM MgCl_2_, 0.2 mM Na_2_GTP, 3 mM Na_2_ATP, 5 mM EGTA, 10 mM Hepes, pH 7.4 (KOH), and 290 mosmol]. Cells were bathed in high potassium extracellular solution (60 mM KCl, 89 mM NaCl, 10 mM Hepes, 1 mM MgCl_2_, 2 mM CaCl_2_, pH 7.4, and 310 mosmol) and GIRK photocurrents were recorded in whole-cell voltage-clamp mode at a holding potential of −75 mV. Light stimulation was delivered using a pE-4000 with properties specified in the figure legends, guided through a 60× water-immersed objective (Olympus). An Axopatch 200B amplifier connected to a DigiData 1440A digitizer was used to control and acquire electrophysiological recordings via Clampex 10.4 (all from Molecular Devices). Data were sampled at 5 kHz, low-pass filtered using a Bessel filter (60 Hz cutoff frequency) and analyzed in Clampfit 11.4.

### Confocal imaging

To assess rhodopsin membrane targeting, HEK293T cells were resuspended under red dim light to polymer-bottom dishes (ibidi, Gräfelfing) 3 to 7 hours after initial transfection, and incubated in the dark at 37°C. At least 12 hours after resuspension, cells were washed twice with imaging buffer [25 mM Hepes and 1.5 g/liter of l-glucose in 50 ml of 10× Hanks’ Balanced Salt Solution (HBSS 10X, Thermo Fisher Scientific)] and maintained at 37°C before acquisition. Samples were imaged using a confocal laser scanning microscope (FV1000, Olympus, Shinjuku, Tokyo, Japan) with a ×60 water immersion objective (UPlanSApo, Olympus). A diode laser set to 559 nm was used to excite mScarlet. The acquired images were analyzed using Fiji (*74*).

### Data processing and statistics

All statistical analyses were performed with GraphPad Prism V10.5.0 software for Windows. Statistical significance for maximal Gq, Gi, and Gs responses was calculated using unpaired one-tailed Student’s *t* tests for normally distributed data and Mann-Whitney *U* for nonnormally distributed data. Hypotheses of normality were tested by the Shapiro-Wilk normality test and homogeneity of variances was tested with the *F* test for *t* tests. A Welch correction was applied when the assumption of homogeneity of variances was rejected. In the figures, the different levels of significance are indicated by *P < 0.05, **P < 0.01, ***P < 0.001, and ****P < 0.0001. Average values are indicated with ±SEM. Light titration curves in the Gq, GsX, and GqX assays were fitted using a dose-response model with a three-parameter sigmoid function, as described previously (*39*).

### Computational modeling of NOM6 and NOL14 conformational ensembles

We aimed to elucidate the inverse spectral properties of NOM6 and NOL14 by analyzing the structural differences between their inactive and active conformational states, corresponding to the 11-*cis* and all-*trans* retinal Schiff base (RSB)–bound forms, respectively. A SPEECH_AF approach (*49*) was used, followed by structural modeling with AlphaFold3 (*48*) to predict protein structures with covalently bound ligands, and evaluate conformations for the inactive and active states. To investigate the effect of the protonation states of NOM6 and NOL14, MD and hybrid QM/MM simulations of NOM6 and NOL14 were then performed as detailed in Supplementary Materials and Methods.

### Calculation of relative sensitivity of individual rhodopsins

Action spectra or absorbance of each expressed rhodopsin was normalized to 1 and multiplied by the transmittance (*T* = 10^−Abs^) of the relevant filter (for distal tiers) or combined filters (for proximal tiers absorbance was additive). Normalization was achieved by dividing all values by the peak sensitivity. Different theoretical absorbance was simulated to show the possible effect of nonuniform filtering on expressed rhodopsins (fig. S15).

## Supplementary Materials

### This PDF file includes:

Supplementary Materials and Methods Figs. S1 to S15 Tables S1 to S5 Legends for data S1 to S6 References
