# Supplementary material for: Spectral tuning and signaling of diverse and far red–sensitive rhodopsins in mantis shrimp eyes
Source: Sci Adv. 2026 Oct 14;12(42):eaef5666. doi: 10.1126/sciadv.aef5666 (PMC13666058; doi:10.1126/sciadv.aef5666)
Supplement: Supplementary file 1 [file sciadv.aef5666_sm.pdf]

Science Advances
AAAS

Supplementary Materials for

Spectral tuning and signaling of diverse and far red–sensitive rhodopsins in mantis shrimp eyes

Camille Brouillon *et al.*

Corresponding author: Alina Pushkarev, alinapushkarev@gmail.com;
Marjorie A. Liénard, marjorie.lienard@uliege.be; Peter Hegemann, hegemann@rz.hu-berlin.de

Sci. Adv. **12**, eaef5666 (2026)
DOI: 10.1126/sciadv.aef5666

This PDF file includes:

Supplementary Materials and Methods
Figs. S1 to S15
Tables S1 to S5
Legends for data S1 to S6
References

Materials and Methods

Cell culture

HEK293T cells (Life technologies) were used for opsin purification, Gq, Gi/o and Gs live bioluminescent second messenger assays and HEK293 lacking GNAS/GNAL/GNAQ/GNA11/GNA12/GNA13/GNAZ (HEK293ΔG7) (*69*) a gift from P. Soba, Friedrich-Alexander-Universität, Erlangen) were used for testing more specifically the Gα signaling selectivity in GsX and GqX assays.

Briefly, cells were grown at 37°C and 5% CO2 in Dulbecco's modified eagle medium (DMEM) supplemented with 10% fetal calf serum (FCS, Biochrome) and 1% penicillin/streptomycin (100µg/ml Biochrome). Two days before measurements, 5 x 10^5 cells/well were seeded in 12-well plates (Sigma-Aldrich). Cells were transiently transfected with a 1:1 ratio of Glo22F luciferase (GloSensor, Promega) for Gi/o, Gs and GsX assays, or aequorin (gift from R. J. Lucas) for Gq and GqX assays, together with opsin plasmid (500 ng/well of each plasmid) using FuGENE HD (Promega) according to the manufacturer's instructions. For GsX and GqX assays, HEK293ΔG7 were also co-transfected with 10ng of individual GsX or GqX chimeras (50:50:1 ratio of GloSensor or Aequorin to opsin to G-protein chimera plasmid). After transfection, all steps were carried out under red dim light. Cells were incubated for 3-7h at 37°C and subsequently resuspended using Trypsin-EDTA (0.05 %, Gibco Thermo Scientific) and transferred to 96 white well-plates (BRAND) in DMEM with 10µM 11-*cis* retinal (gift from Lisa Neuhold and Clint Markino, National Eye Institute, National Institutes of Health). At least 12 h after resuspension, the medium was exchanged with transparent Leibovitz's L-15 medium with 10% FCS. For Gq and GqX assays, L-15 medium was supplemented with Coelenterazine-h (10µM, Promega) and cells were incubated for 2h at Room Temperature (RT). For Gs, Gi/o and GsX assays cells were incubated in L-15 medium with beetle luciferin (2 mM in 10 mM HEPES pH 6.9, Promega) for 1h at RT.

NOL opsin purification for dark absorbance spectra measurements

HEK293T cells were cultured in DMEM with high glucose (GIBCO) supplemented with 10% fetal bovine serum (FBS, Life Technologies) without antibiotics. For each purification, twenty 10-cm dishes were seeded with HEK293T cells at 2M on day 1 and transfected 24 hours later with lipid complexes formed in serum-reduced Opti-MEM (Life Technologies) with 24 µg Endotoxin-free plasmid DNA (pcDNA5-FLAG-T2A-Sirius) (fig. S4A) and 72 µL Polyethylenimine (1 mg/mL in pure water; PEI 25,000MW, PolySciences). 11-*cis*-retinal was added to plates at 5µM (in 100% Ethanol) under dim red light illumination 6h post transfection together with fresh culture medium, then plates were wrapped in aluminium and placed back in the incubator. One transfected plate was wrapped separately as control for fluorescence visualization. Forty eight hours after transfection, the medium was discarded, cells were scraped and collected in a a black tube in a total volume of 50 mL Hepes cold resuspension buffer containing 3 mM $MgCl_2$, 50 mM Hepes pH8.5, 140 mM NaCl and EDTA-free protein inhibitors (Sigma-Aldrich), washed, centrifuged for 10 min at 4°C and 1,620 x g and resuspended in 10 ml cold resuspension buffer then nutated for 90 min with 40 µM 11-*cis*-retinal and 10 rpm rotation. Following ultracentrifugation at 21,500 rpm for 25 min, the total membrane fraction was extracted in cold solubilization Hepes buffer (3 mM $MgCl_2$, 50 mM Hepes pH8.5, 140 mM NaCl) supplemented with 1% DDM and 25% Glycerol vol/vol for 1h at 4°C with 10 rpm

rotation cycles. The crude protein extract (supernatant) was collected after ultracentrifugation for 20 min at 21,500 rpm and the opsin-FLAG fraction was bound in a 15-mL black tube to 1000 µL Pierce Anti-DYKDDDDK Affinity Resin (50% slurry, Thermo Scientific) at 4°C overnight under gentle rotation. The next day, the resin-bound fraction was loaded on a 5 mL Pierce column, washed 3x by inversion in 3mL cold wash Hepes buffer (3 mM $MgCl_2$, 50 mM Hepes pH 8.5, 140 mM NaCl) containing 25% Glycerol vol/vol, 0.1% DDM, without protease inhibitors, followed by centrifugation for 1 min at 1,000 x g at 10°C to collect wash fractions. Active opsin complexes in solution were eluted for 20 min at room temperature and 10 rpm rotation, via competitive binding with 150 µL FLAG peptide diluted in wash Hepes buffer (5 mg/mL). After centrifugation for 1 min at 1,000 x g, the total eluate fraction was concentrated for 1 hour at 4°C and 4,000 rpm using an Amicon Ultra-2 filter column to a final volume of approx. 200 µL, divided in 40-50 µL aliquots in 1.5mL black tubes and stored on ice in the dark. Under dim-red light, 1.5µL-aliquots were loaded on a Nanodrop1000 (Life technologies) to record UV-Vis spectra taken at 5-nm intervals from 200 to 750 nm.

Computational modeling of NOM6 and NOL14 conformational ensembles

We aimed to elucidate the inverse spectral properties of NOM6 and NOL14 by analyzing the structural differences between their inactive and active conformational states, corresponding to the 11-*cis* and all-*trans* retinal Schiff base (RSB) bound forms, respectively. Because experimental structures were unavailable, we employed the SPEACH_AF approach (*49*), which generates multiple conformational states through systematic alanine scanning of the multiple sequence alignment (MSA), followed by structural modeling with ColabFold (*75*) using the modified MSA and subsequent model filtering using MolProbity (*76*). In this study, we replaced ColabFold with AlphaFold3 (*48*), which allows prediction of protein structures in complex with covalently bound ligands. As AlphaFold3 employs MSAs generated by JackHMMER 3.4 (*77*), we used these alignments instead of those from MMseqs2 (*78*), given that both share the same A3M format. To focus on evolutionally conserved positions rather than insertions specific to individual sequences, only conserved residues (uppercase letters in A3M) were retained. For this purpose, the SPEACH_AF notebook code was modified from if temp[n] != '-': to if temp[n] != '-' and temp[n].isupper():.

To increase the number of conformational samples, we applied a sliding window size of five for alanine substitutions and used a distance cutoff of 4 Å for alanine substitutions of adjacent residues, based on the initial structural model. This process produced 64 and 66 MSAs for NOM6 and NOL14, respectively, and 15 structures per MSA were generated using three random seeds. The resulting conformations were evaluated with MolProbity (*76*), as implemented in Phenix 1.21 (*79*) for structural quality assessment. Mean MolProbity scores were calculated for each set, and those exceeding one standard deviation above the mean were discarded. This procedure yielded 885 and 810 conformations for NOM6 and NOL14, respectively. Because these conformations included alanine mutations introduced during MSA-based sampling, we reverted the mutated residues to the wild-type sequences using MODELLER 10.1 (*80*). To model the 11-*cis* and all-*trans* RSB binding modes more accurately, the predicted retinal moiety was removed and redocked using GOLD software 2024.3 (*81*) with retinal coordinates from PDB entries 2Z73 (*82*) (11-*cis*) or 8A6E (*83*) (all-*trans*). Conformations with negative GOLD fitness scores were excluded, resulting in 843 and 776 conformations for the 11-

cis retinal bound states of NOM6 and NOL14, respectively, and 823 and 758 for the all-*trans* retinal bound states. To identify models representing the inactive and active states, we selected the 11-*cis* RSB-bound (inactive-like) and the all-*trans* RSB-bound (active-like) conformations based on principal component analysis (PCA) of Cα atoms excluding the flexible N- and C-termini (M1-N57 and M357-A385 in NOM6; M1-D45 and L343-A372 in NOL14) using ProDy 2.5 (*84*), visual inspection of key motif conformations, and GOLD fitness scores (figs. S9 and S10). These selected models were subsequently subjected to molecular dynamics (MD) simulations to remove nonphysical contacts and equilibrate the rhodopsins within lipid and solvent environments.

Molecular dynamics and hybrid quantum mechanics / molecular mechanics simulations

Molecular dynamics (MD) simulations of NOM6 and NOL14 were performed using GROMACS 2023 (*85*) with CHARMM36m force field (*86*). To investigate the effect of the protonation states of D234 in NOM6 on spectral shifts, MD simulations were carried out for both the deprotonated and protonated forms of D234 in the 11-*cis* and all-*trans* retinal Schiff bound states. Each rhodopsin model was embedded in a bilayer of 1-palmitoyl-2-oleoyl-sn-glycero-3-phosphocholine (POPC) lipids and solvated with TIP3 water model (*87*) containing 150 mM NaCl, using the CHARMM-GUI webserver (*88*) (table S3). Force field parameters for the RSB were taken from previously published work (*89–91*). Energy minimization and equilibration were performed with a stepwise release of positional and angular restraints on the RSB, protein, and lipids, as summarized in table S4. The equilibrated systems were then used for three independent 500-ns production MD simulations at temperature of 303 K. A short-range cutoff of 12 Å was applied for both electrostatic and van der Waals interactions, while long-range electrostatics were treated using the particle mesh Ewald method (*92*). Temperature and pressure were maintained at 303 K and 1 bar, respectively, using velocity rescaling (*93*) for temperature control and either Berendsen (*94*) or Parinello-Rahmann (*95*) coupling for pressure control. Following the simulations, conformational clustering was performed using backbone atoms of the transmembrane helices (ClusterBB) and residues in the retinal binding pocket within a cutoff of 4.5 Å (ClusterRBP) (table S5). Clustering was carried out using the GROMOS algorithm (*96*), with root mean square deviation (RMSD) cutoffs of 2 Å and 1.3 Å for ClusterBB and ClusterRBP, respectively. Representative structures from each cluster were selected for subsequent analyses.

To compute electronic properties of RSB in the protein environment, hybrid quantum mechanics / molecular mechanics (QM/MM) simulations were conducted using Amber2021 (*97*) with the second-order density functional tight binding (DFTB2) method (*98*) including dispersion correction (*99*). The RSB was designated as the QM region by severing Cα-Cβ bond and capping it with a hydrogen link atom, while the remaining system was treated with classical force fields. Energy minimization was performed using 2,500 steps of steepest descent followed by up to 2,500 steps of conjugate gradient minimization with positional restraints of 10 kcal/mol/Å2 on heavy atoms. This was followed by QM/MM equilibration, consisting of a gradual temperature increase from 0 to 303 K, 50 ps of dynamics at 303 K, and a 1-ns production simulation at 303 K using a Langevin thermostat under constant volume, a 1-fs time step, and a 12 Å cutoff for electrostatic and van der Waals interactions. Spectroscopic properties were computed from the QM/MM trajectories by extracting snapshots every 10 ps (100 snapshots in total) and calculating vertical excitation energies using the second-order algebraic diagrammatic

construction [ADC(2)] method (*100*) with the correlation-consistent polarized valence double-zeta (cc-pVDZ) basis set in TURBOMOLE 7.7 and 7.8 (*101*).

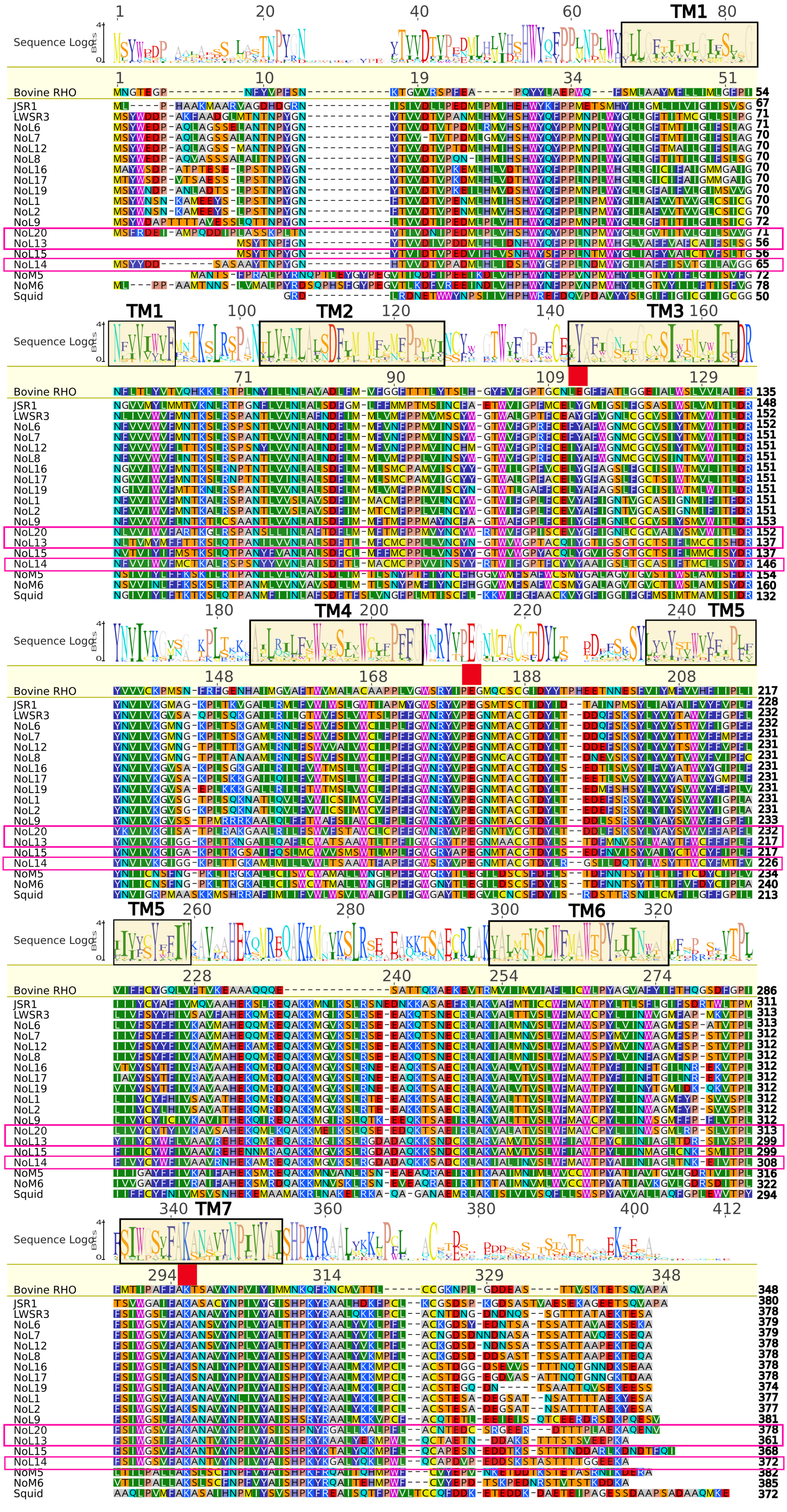

Sequence Logo
TM1
TM2
TM3
TM4
TM5
TM6
TM7
Bovine RHO
JSR1
LWSR3
NoL6
NoL7
NoL12
NoL8
NoL16
NoL17
NoL19
NoL1
NoL2
NoL9
NoL20
NoL13
NoL15
NoL14
NoM5
NoM6
Squid

Fig. S1. Comparison of the full length NOL and NOM sequences of *N. oerstedii*.

Alignment with squid, jumping spider rhodopsin 1 mapped to bovine rhodopsin for reference. (PDB: 2ZIY, 6I9K and Uniprot: P02699 respectively) by using MAFFT alignment tool (*102*). Predicted common transmembrane helices are marked with black rectangles. The most red sensitive rhodopsins are marked with pink rectangles. Sequences were aligned using and visualized using the RasMol amino-acid color scheme, in which residues are grouped by physicochemical properties. The counterion of vertebrates at position 113, of invertebrates at pos. 181, and retinal-binding Lys at position 296 are labelled by red squares.

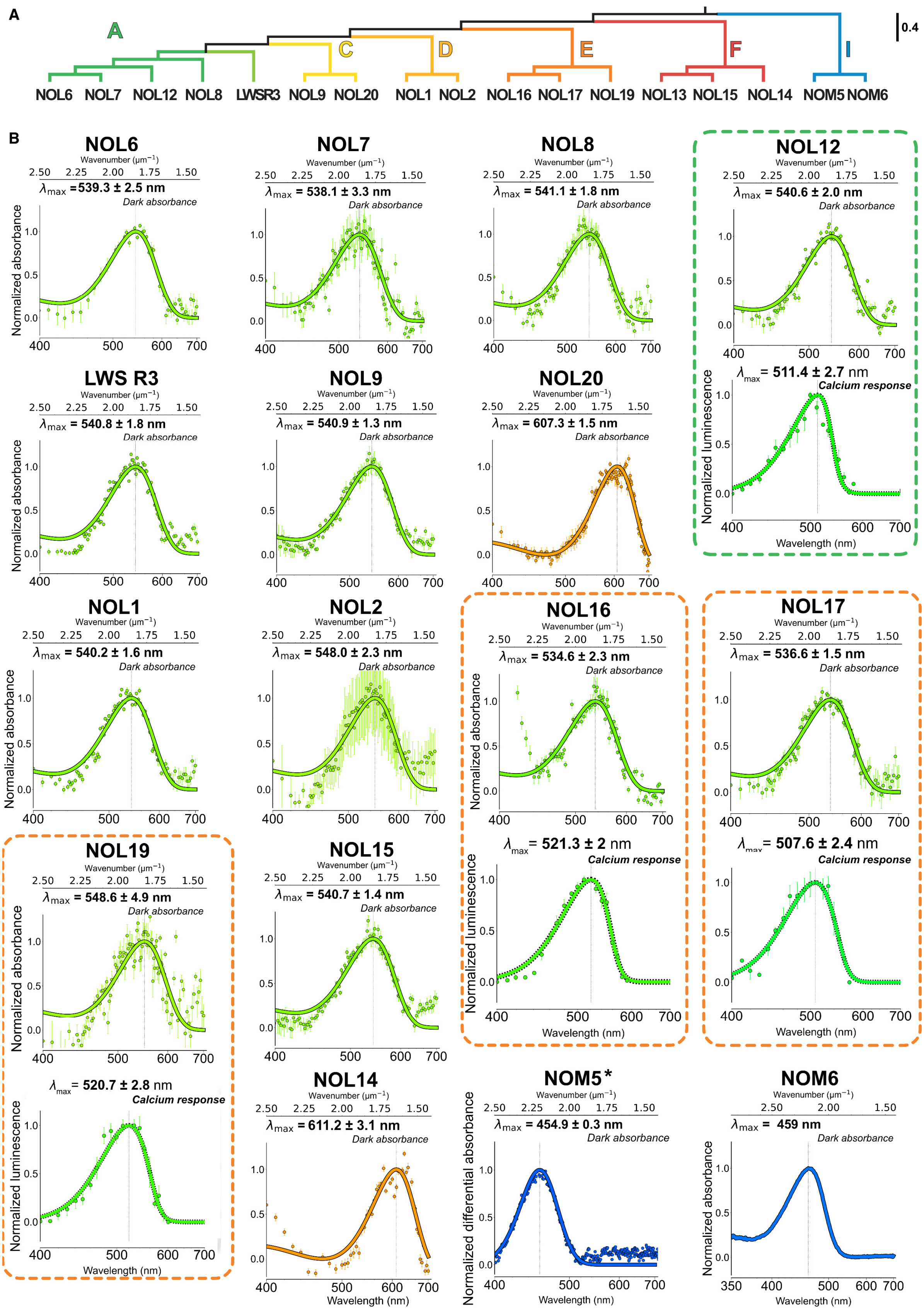

A
B
NOL6 NOL7 NOL12 NOL8 LWSR3 NOL9 NOL20 NOL1 NOL2 NOL16 NOL17 NOL19 NOL13 NOL15 NOL14 NOM5 NOM6
C
D
E
F
I
0.4
NOL6
λmax =539.3 ± 2.5 nm
NOL7
λmax = 538.1 ± 3.3 nm
NOL8
λmax = 541.1 ± 1.8 nm
NOL12
λmax = 540.6 ± 2.0 nm
λmax = 511.4 ± 2.7 nm
Calcium response
LWS R3
λmax = 540.8 ± 1.8 nm
NOL9
λmax = 540.9 ± 1.3 nm
NOL20
λmax = 607.3 ± 1.5 nm
NOL1
λmax = 540.2 ± 1.6 nm
NOL2
λmax = 548.0 ± 2.3 nm
NOL16
λmax = 534.6 ± 2.3 nm
λmax = 521.3 ± 2 nm
Calcium response
NOL17
λmax = 536.6 ± 1.5 nm
λmax = 507.6 ± 2.4 nm
Calcium response
NOL19
λmax = 548.6 ± 4.9 nm
λmax = 520.7 ± 2.8 nm
Calcium response
NOL15
λmax = 540.7 ± 1.4 nm
NOL14
λmax = 611.2 ± 3.1 nm
NOM5*
λmax = 454.9 ± 0.3 nm
NOM6
λmax = 459 nm
Dark absorbance
Wavenumber (μm⁻¹)
Wavelength (nm)
Normalized absorbance
Normalized luminescence
Normalized differential absorbance

Fig. S2. Dark absorption and action spectra of NOL and NOM opsins.
(A) Phylogenetic relations of NOLs and NOMs from clade A, C, D, E, F and I (adapted from Porter et al. (*28*) with permission). (B) Dark absorbance spectra measured in an UV-vis Nano-spectrometer after reconstitution with 11-*cis* retinal, with corresponding action spectra below when possible, established from maximal aequorin responses, as indicated by the dashed frames, color coded according to phylogenetic branch. Light intensity of the 1 s pulse used for the action spectra: NOL12: $8x10^{14}$ photons cm^{-2} s^{-1} (n=4); NOL16 (n=3) and NOL17 (n=3): $4x10^{14}$ photons cm^{-2} s^{-1}; NOL19: $8x10^{13}$ photons cm^{-2} s^{-1} (n=4). Absorbance and action spectra of all NOLs were fitted with a non-linear least squares optimization of the Govardovskii visual template to estimate the best-fit λmax. Data is presented as mean values and with ± SEM for NOLs. For NOL6 and NOL 14 spectra are redrawn from raw absorbances previously reported (*103*). Please note that the NOL 14 λmax as fitted here does not decompose its two identified spectral confirmations (fig. S8). For NOLs, the mean absorbance maxima (O.D.) ± SD (10 mm path) across 1.5 µl rhodopsin measurements were as follows: NOL1 (n=6; OD= 0.10±0.045), NOL2 (n=3, OD=0.08±0.079), NOL6 (n=9, OD=0.16±0.03), NOL7 (n=11, OD= 0.08±0.05), NOL8 (n=11, OD=0.067±0.05), NOL9 (n=6, OD=0.21±0.16), NOL12 (n=9, OD=0.19±0.143), NOL14 (n=14, OD=0.177±0.09), NOL15 (n=8, OD=0.167±0.06), NOL16 (7, OD=0.146±0.036), NOL17 (n=5, OD=0.112±0.05), NOL19 (n=6, OD=0.108±0.033), NOL20 (n=9, OD=0.23±0.17), LWSR3 (n=11, OD=0.102±0.04), where n represents the number of measurements with active rhodopsin complexes. For NOM6 and NOM5, spectra were recorded in 50 µl volume, with mean OD at λmax of 0.138 and 0.0993, respectively.
Dark absorption for NOM5 was measured with 9-*cis* retinal (*) and plotted as the differential absorbance between the dark absorption and the absorption after a 60s pulse of light (455 nm), fitted with a simple gaussian. For NOM6 a continuous spectra without any fitting but corrected for Mie scattering is shown. The dark absorption spectrum of NOL13 is not reported due to the instability of the rhodopsin in solution; its activation wavelength was recorded in live HEK cells (Fig. 2B). Bimodal action spectra for NOL13, NOL14, NOM5 and NOM6 are presented in Fig. 2 C.

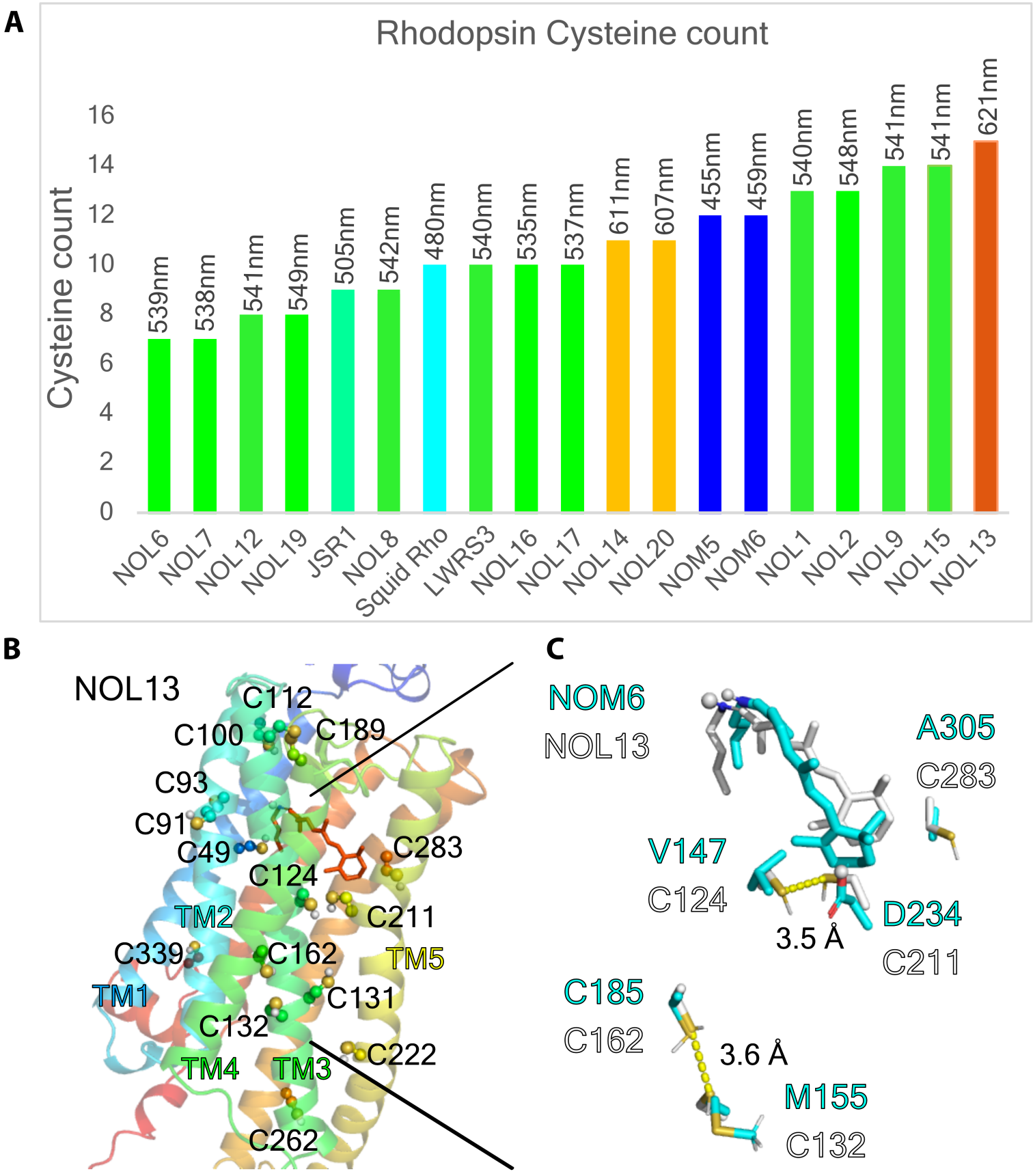

Fig. S3. Cysteine mapping of expressed rhodopsins.

(A) Number of Cys in the different rhodopsins. (B) Location of the Cys in NOL13 (C) Possible cystine bridges at the ring site of NOL13 and NOM6.

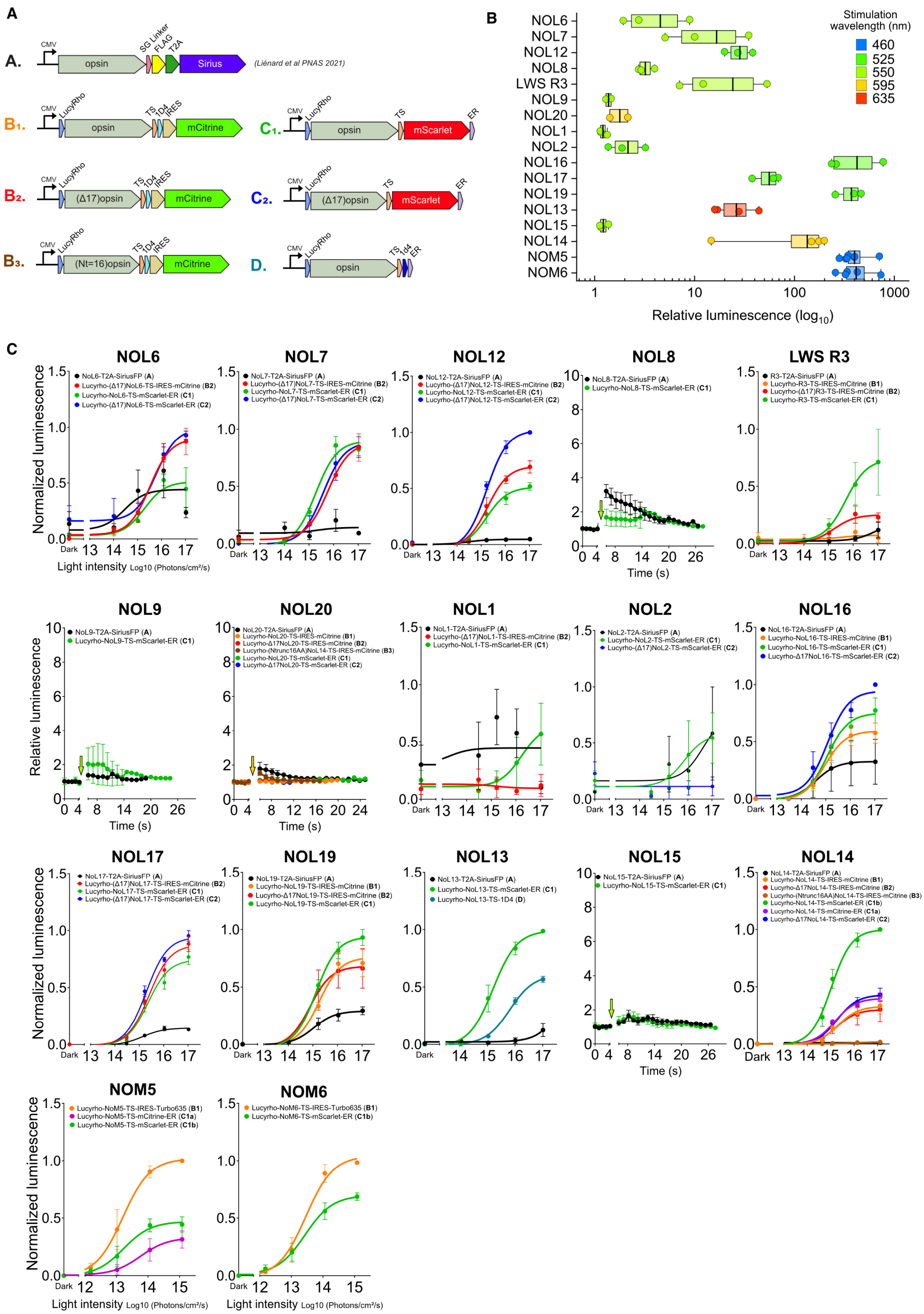
A
B
C
A.
B1.
B2.
B3.
C1.
C2.
D.
CMV
LucyRho
opsin
(Δ17)opsin
(Nt=16)opsin
mCitrine
mScarlet
Sirius
SG Linker
FLAG
T2A
TS
1D4
IRES
ER
(Liénard et al PNAS 2021)
Stimulation wavelength (nm)
460
525
550
595
635
NOL6
NOL7
NOL12
NOL8
LWS R3
NOL9
NOL20
NOL1
NOL2
NOL16
NOL17
NOL19
NOL13
NOL15
NOL14
NOM5
NOM6
Relative luminescence (log10)
Normalized luminescence
Relative luminescence
Light intensity Log10 (Photons/cm²/s)
Time (s)
Dark

Fig. S4. Optimization of constructs.

(A) Design of DNA constructs used for optimisation of the luminescent responses (SG linker 2aa, 1D4 affinity tag TETSQVAPA 9aa, FLAG affinity tag DYKDDDDK 8aa, LucyRho self-cleavable leucine-rich signal peptide, T2A Self cleaving peptide from *Thosea asigna* virus 2A GSGEGRGSLLTCGDVEENPGP 21aa, TS Golgi export trafficking signal sequence KSRITSEGEYIPLDQIDINV 20aa, ER endoplasmic reticulum export sequence FCYENEV 7aa, IRES internal ribosome entry site, Δ17 deletion of t17aa of an opsin N-terminus, Nt=16 only 16aa remaining upstream of TM1 of the opsin). Construct A. has been previously described (*103*). (B) Comparison of relative aequorin luminescent levels of the most effective (highest signal) constructs for each opsin following a 1s activation (wavelength indicated in the legend). (C) Light-titration aequorin responses of various DNA constructs used in this study. Unresponsive opsins (NOL8, NOL9, NOL15, NOL20) signals are shown as relative luminescence over time, at the highest light intensity ($1x10^{17}$ photons cm^{-2} s^{-1}).

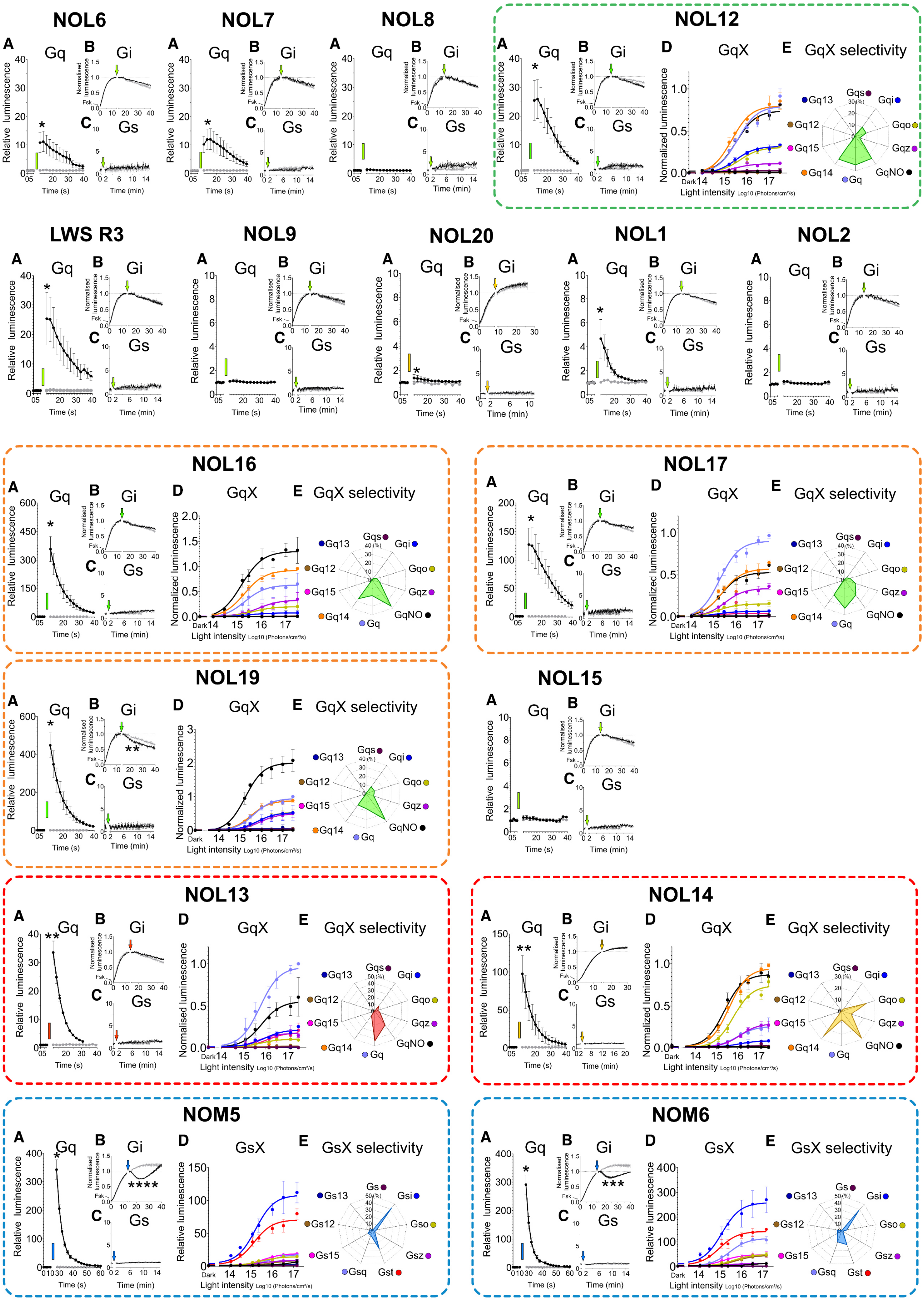
NOL6
NOL7
NOL8
NOL12
LWS R3
NOL9
NOL20
NOL1
NOL2
NOL16
NOL17
NOL19
NOL15
NOL13
NOL14
NOM5
NOM6
A Gq
B Gi
C Gs
D GqX
E GqX selectivity
D GsX
E GsX selectivity
Relative luminescence
Normalised luminescence
Normalized luminescence
Time (s)
Time (min)
Fsk
Light intensity Log10 (Photons/cm²/s)
Dark
Gq13
Gq12
Gq15
Gq14
Gq
GqNO
Gqz
Gqo
Gqi
Gqs
Gs13
Gs12
Gs15
Gsq
Gst
Gsz
Gso
Gsi
Gs

Fig. S5. G-protein selectivity profiles of NOL and NOM opsins.

(A) Responses to the Gq-protein family were measured by using the aequorin assay (see table S1 for detailed values). (B-C) Coupling efficiency to the (B) Gi- and (C) Gs-protein families in the glosensor assay. Relative luminescence values are shown as the raw luminescence divided by the averaged pre-illumination baseline. For Gi, cAMP luminescent levels were elevated using 2μM forskolin, 15 min before illumination. (D) Light-dependent G-protein signaling was further investigated for NOMs using the GsX assay and for NOLs using the GqX assay when possible indicated by dashed frames, colourized based on their phylogenetic clade (fig. S2A). Light-dependent maximal post-stimuli responses are normalized between the no GqX/GsX control and the maximal relative response. (E) Selectivity profiles were established from maximal responses to GsX or GqX chimeras and expressed as percentages of the sum of GsX or GqX protein activity. NOL12, NOL16, NOL17, NOL19 were stimulated with 525 nm light, NOL6, NOL7, NOL8, NOL9, NOL1, NOL2, NOL15, LWS R3 with 550 nm, NOL14, NOL20 with 595 nm, and NOL13 with 635 nm. All responses were measured after a dark pre-illumination baseline followed by a 1s light pulse, indicated by colored arrows and rectangles. All data is presented as mean ± SEM. Different levels of significance at the peak are indicated by * for $p < 0.05$, ** for $p < 0.01$, *** for $p < 0.001$, and **** for $p < 0.0001$, compared to cells transfected only with luminescent reporters (grey traces).

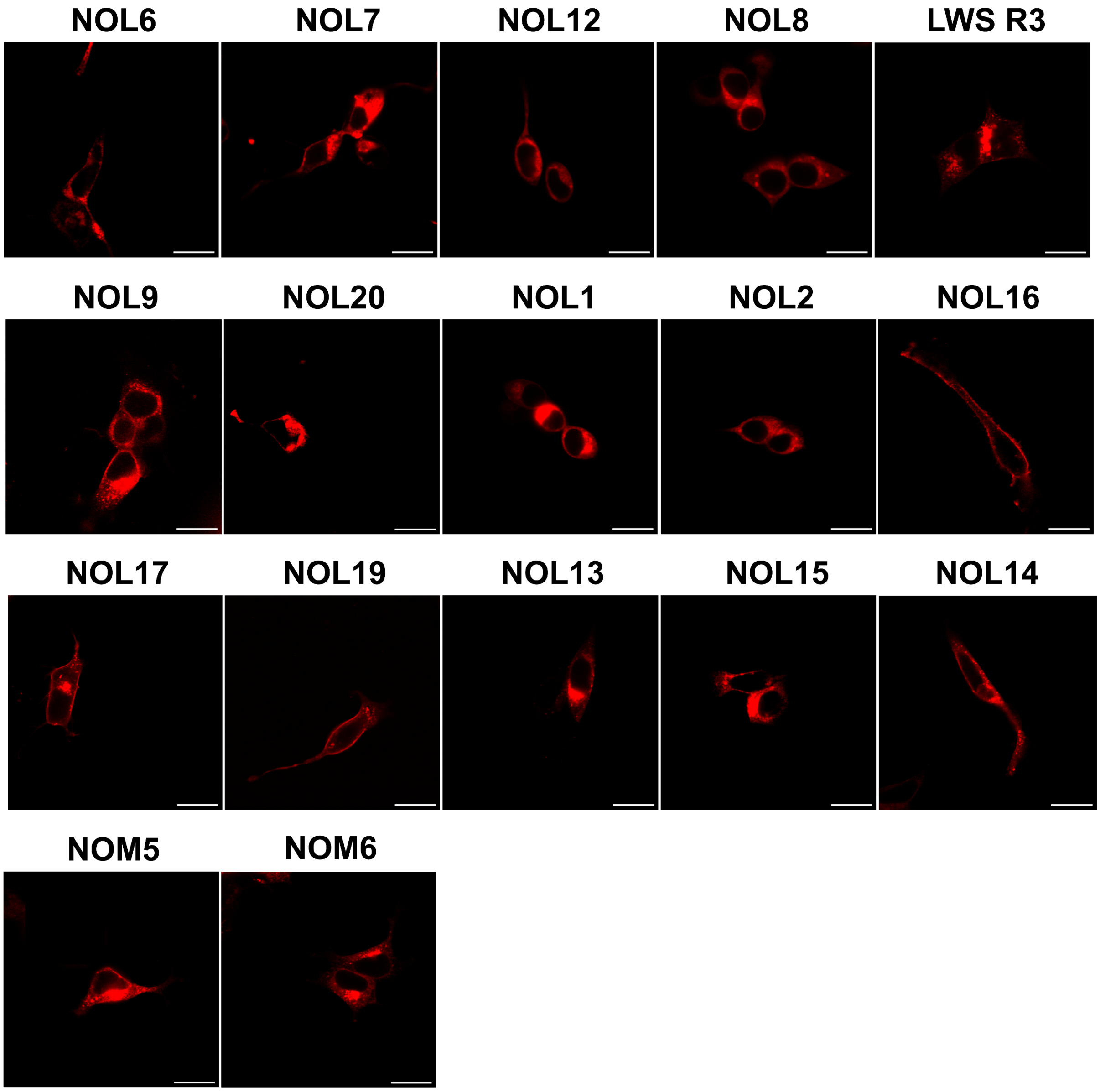

Fig. S6. Expression profile of NOM and NOL rhodopsins.
HEK293T cells expressing NOM or NOL opsins fused to mScarlet at the C-termini in construct C1 (fig. S4). Scale bar = 20µm.

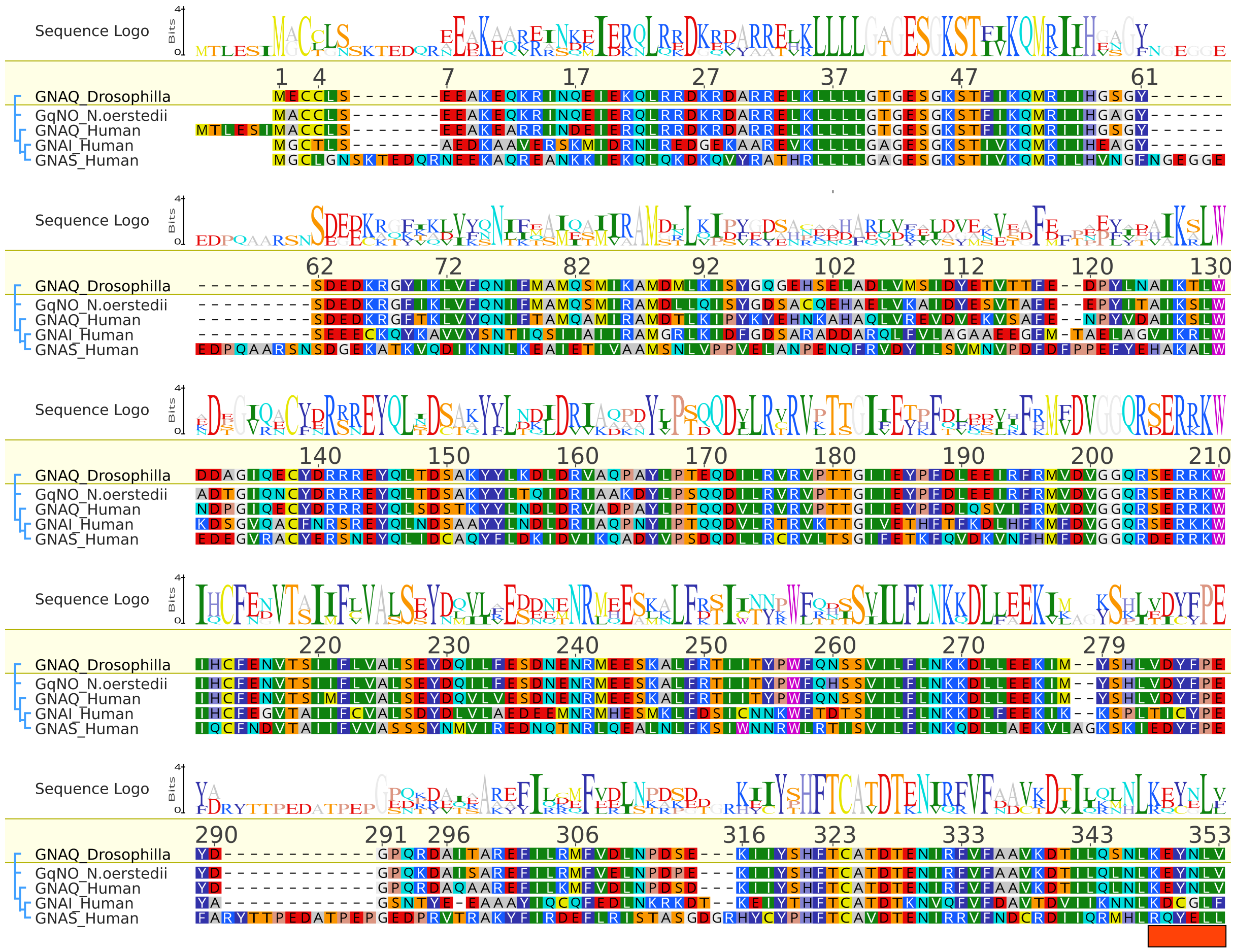

Fig. S7. Alignment of native Gαq from *N. oerstedii* (GqNO) and human Gαq, Gαs, Gαi and invertebrate *Drosophila melanogaster* Gαq canonical splice variant for reference. Multiple sequence alignments were generated using MAFFT *v7.450 (102)* and FastTree 2.1.11 was used to generate the phylogeny in blue on the left. The critical α5 helix is marked with an orange rectangle. Sequences colorized according to the RasMol amino-acid color scheme, in which residues are grouped by physicochemical properties.

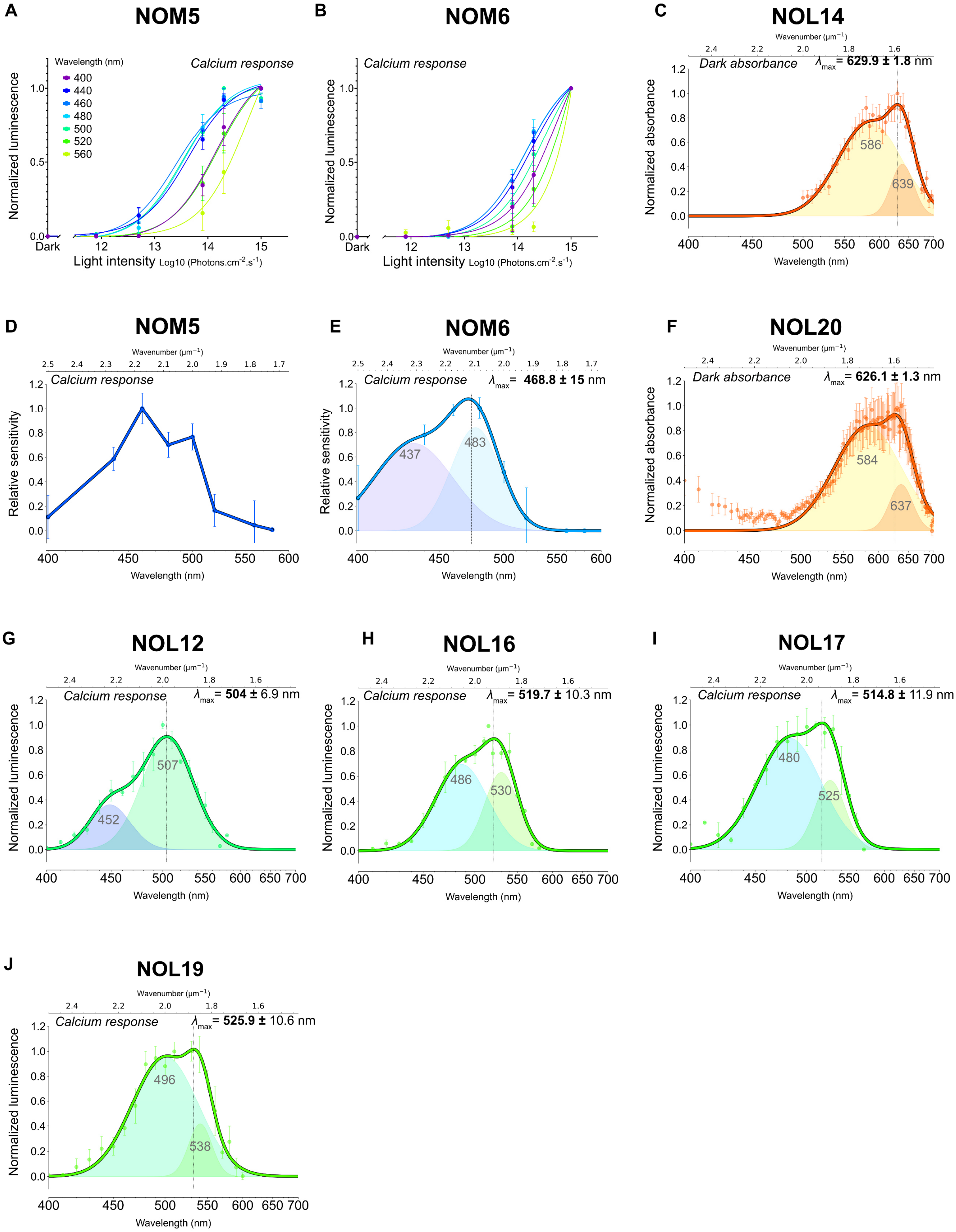

A
NOM5
Calcium response
Wavelength (nm)
400
440
460
480
500
520
560
Normalized luminescence
Light intensity Log10 (Photons.cm-2.s-1)
Dark
B
NOM6
Calcium response
Normalized luminescence
Light intensity Log10 (Photons.cm-2.s-1)
Dark
C
NOL14
Wavenumber (μm−1)
Dark absorbance
λmax = 629.9 ± 1.8 nm
586
639
Normalized absorbance
Wavelength (nm)
D
NOM5
Wavenumber (μm−1)
Calcium response
Relative sensitivity
Wavelength (nm)
E
NOM6
Wavenumber (μm−1)
Calcium response
λmax = 468.8 ± 15 nm
437
483
Relative sensitivity
Wavelength (nm)
F
NOL20
Wavenumber (μm−1)
Dark absorbance
λmax = 626.1 ± 1.3 nm
584
637
Normalized absorbance
Wavelength (nm)
G
NOL12
Wavenumber (μm−1)
Calcium response
λmax = 504 ± 6.9 nm
452
507
Normalized luminescence
Wavelength (nm)
H
NOL16
Wavenumber (μm−1)
Calcium response
λmax = 519.7 ± 10.3 nm
486
530
Normalized luminescence
Wavelength (nm)
I
NOL17
Wavenumber (μm−1)
Calcium response
λmax = 514.8 ± 11.9 nm
480
525
Normalized luminescence
Wavelength (nm)
J
NOL19
Wavenumber (μm−1)
Calcium response
λmax = 525.9 ± 10.6 nm
496
538
Normalized luminescence
Wavelength (nm)

Fig. S8. **Dark state spectral composition of NOM5, NOM6, NOL12, 14, 16, 17, 19, and 20.** Light-dependent aequorin responses following a 1s light pulse of (A) NOM5 and (B) NOM6 normalized to the maximal luminescent response per wavelength (n=3). Spectral relative sensitivity of (D) NOM5 and (E) NOM6 calculated from EC50 values extracted from (A) and (B) at each wavelength. (C) Dark absorbance spectrum of NOL14 replotted from (*103*) and of (F) NOL 20, and (G-J) Dark luminescence spectrum of NOL12, 16, 17 and 19 refitted from fig. S2. NOL12, 14, 16, 17, 19, and 20 spectra are fitted with a two component Gaussian Mixture Model (GMM) suggesting the existence of two conformationally different isoforms. In E-I representations, the highest maximal wavelength value is indicated above the average peak (dashed vertical line). Data is presented as mean ± SEM.

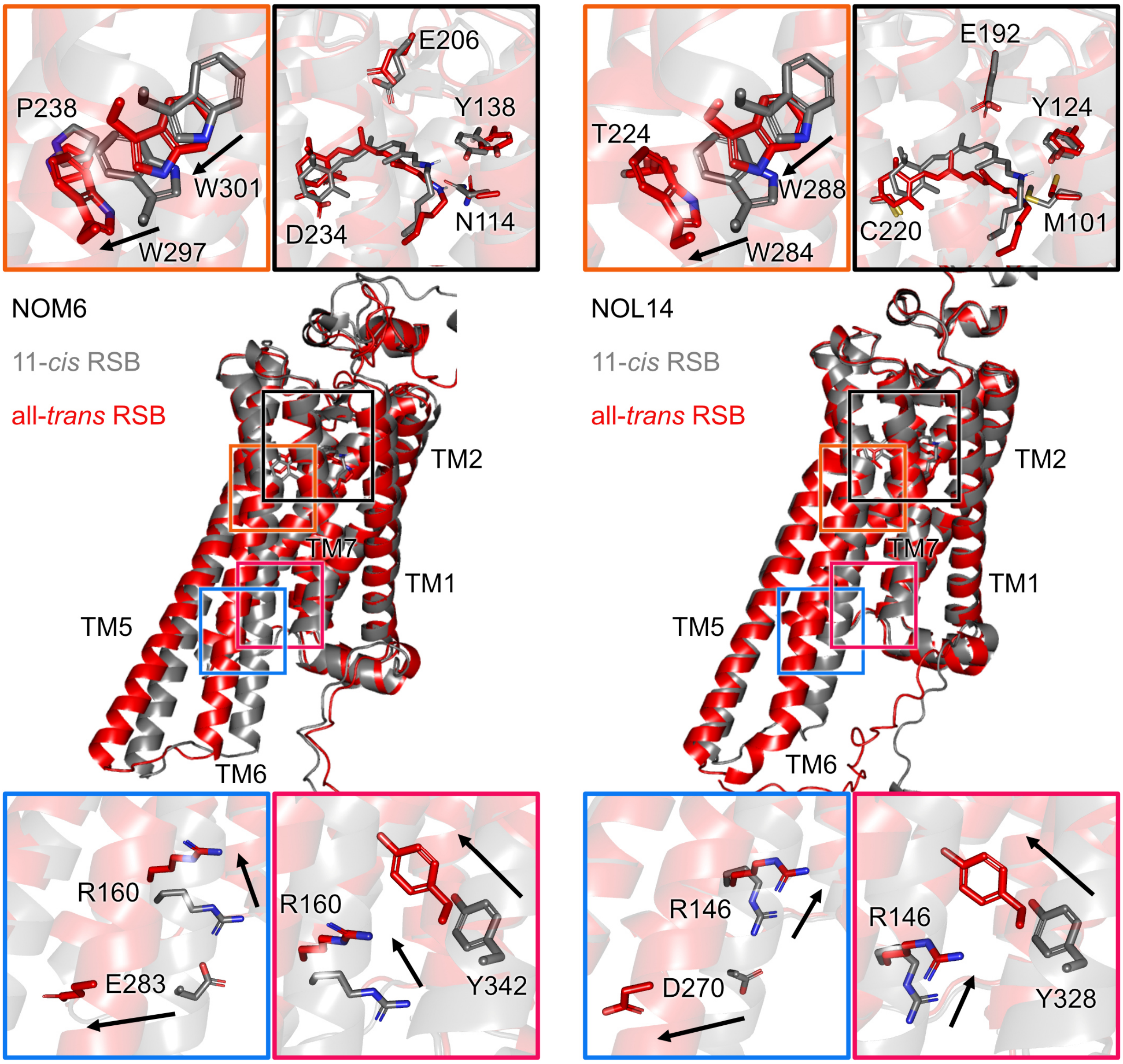

Fig. S9. Comparison of computed structural models between NOM6 and NOL14.

Inactive (11-*cis*-retinal Schiff base bound) and active (all-*trans* retinal Schiff base bound) states are shown as gray and red cartoons, respectively. In the active states, conserved motifs undergo similar rearrangement that distinguish them from the inactive states (*104*). The PIF motif contains a tryptophan substitution at position 6.44 adjacent to the CWxP tryptophan. Activation rearranges these residues (W297/W301 in NOM6 and W284/W288 in NOL14), reshaping the hydrophobic core, disrupting the DRY ionic lock (R160-E283 and R146-D270), and reorienting the NPxxY tyrosine (Y342 and Y328) toward the intracellular cavity.

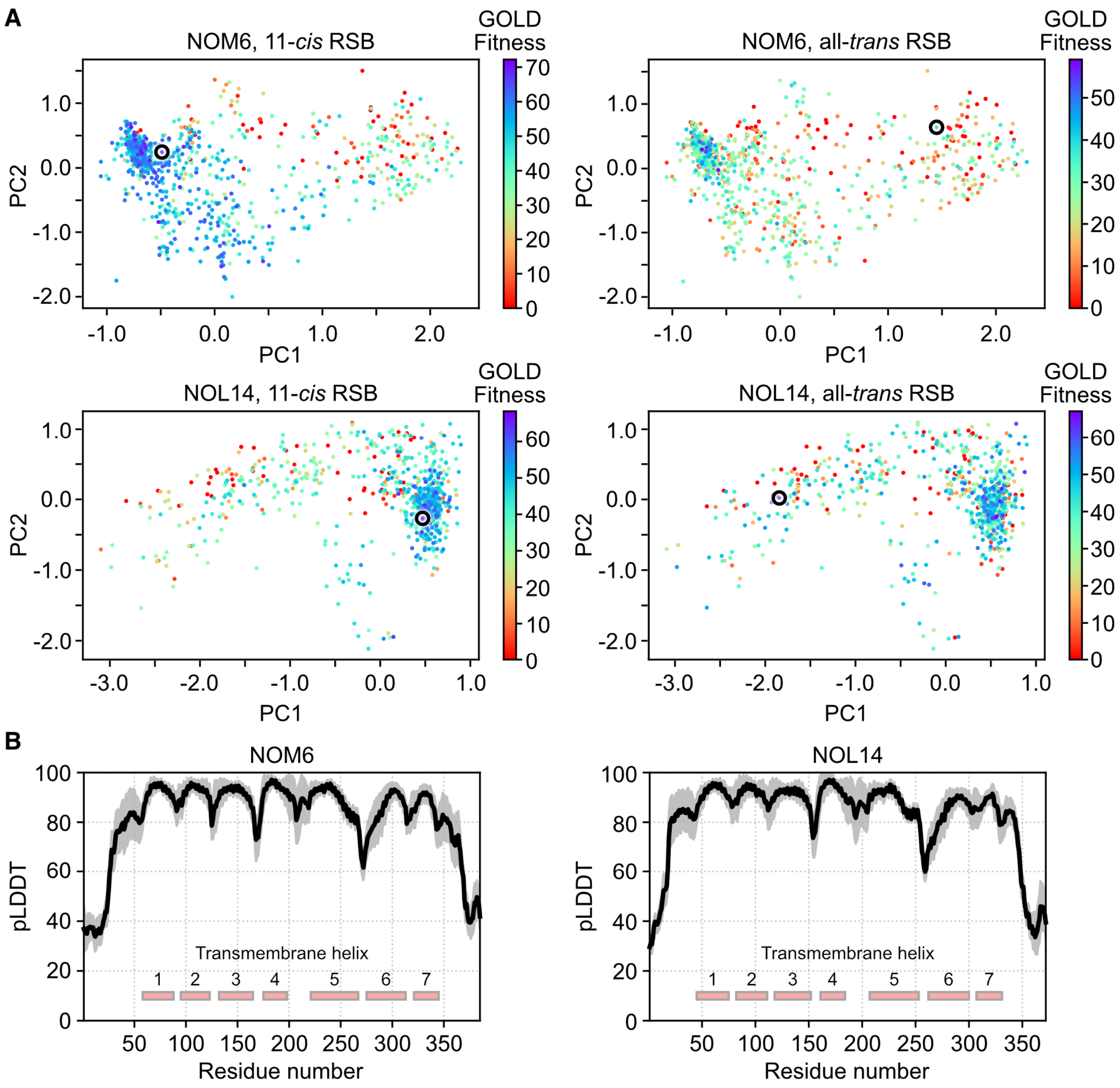

Fig. S10. Predicted conformational ensembles and evaluation of model accuracy.

(A) Principal component analysis (PCA) of NOM6 and NOL14 conformations, excluding the N- and C-terminal regions. Docking scores for the 11-*cis* and all-*trans* retinal Schiff bases are shown by color coding, and the conformations with negative scores were discarded. Selected inactive-like (left) and active-like (right) states are indicated by black circles. (B) Predicted local distance difference test (pLDDT) scores of the conformations. Transmembrane helices are shown as red rectangles. The black line represents the mean pLDDT of Cα atoms, and the gray shading indicates the standard deviation. The number of conformations analyzed was 885 for NOM6 and 810 for NOL14.

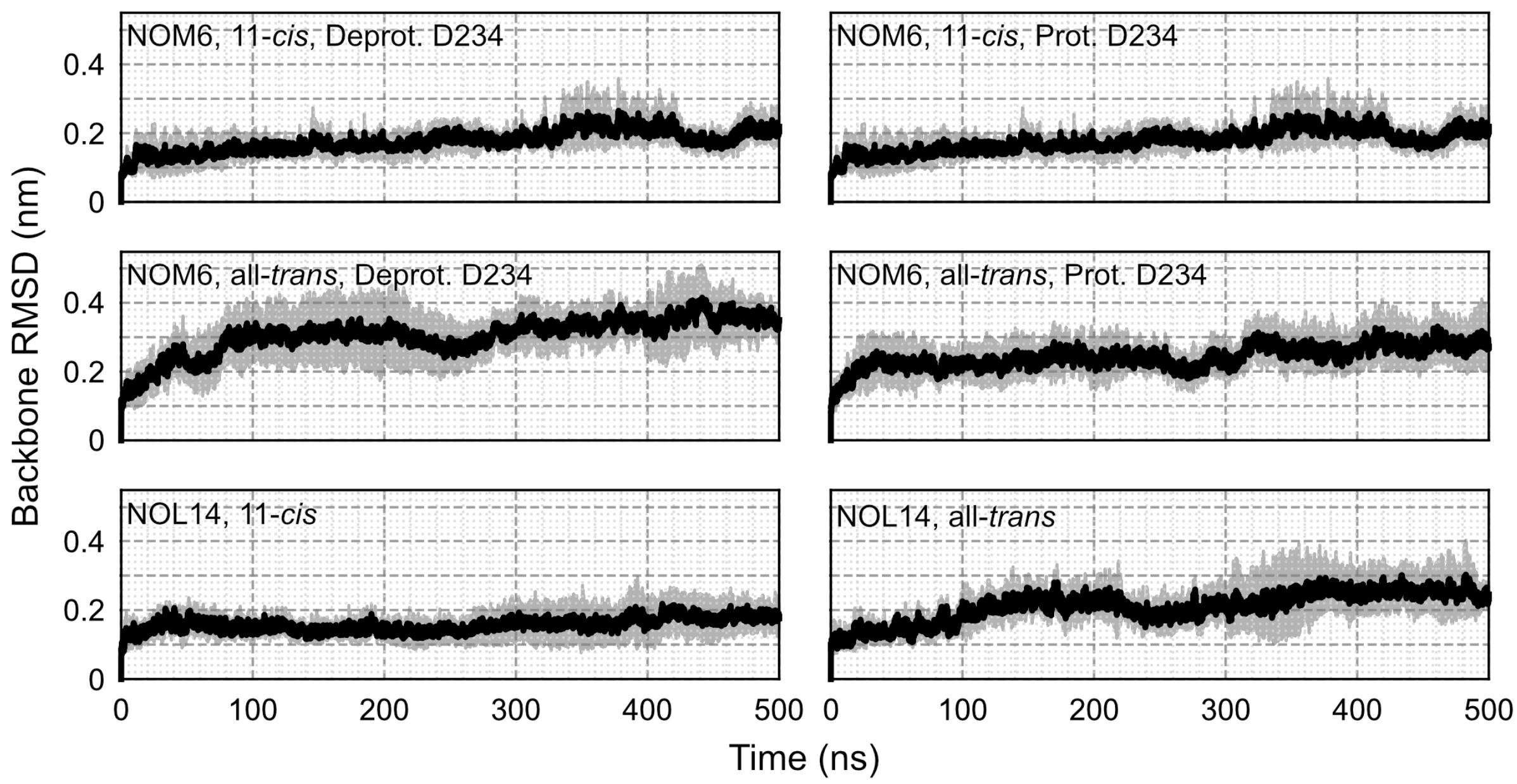

Fig. S11. Root mean square deviations (RMSDs) of NOM6 and NOL14.

Backbone RMSDs were calculated excluding the N- and C-terminal regions. The solid line represents the mean RMSD, and the shaded area indicates the standard deviation. Molecular dynamics simulations were performed for 500 ns and repeated three times.

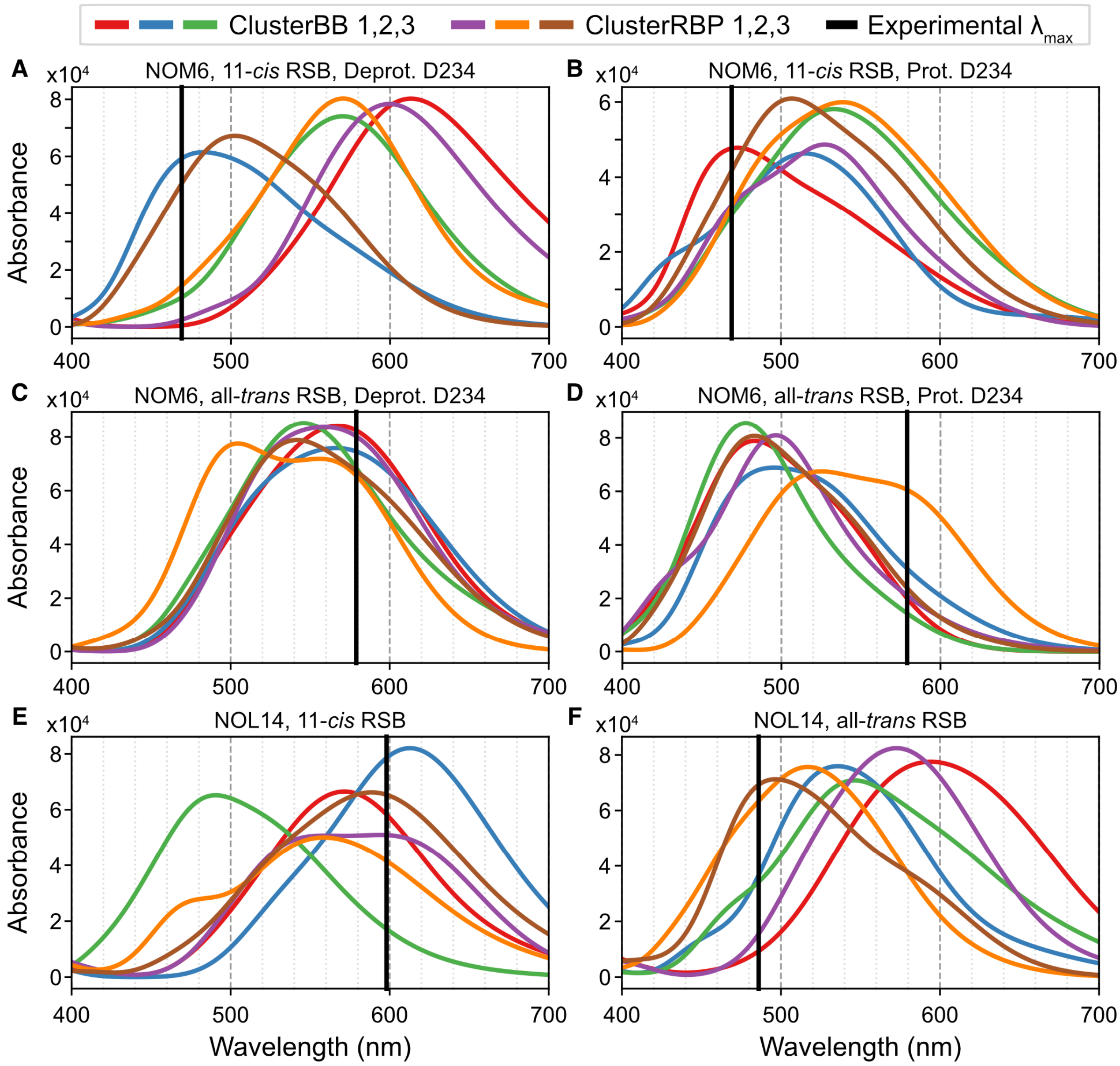

Fig. S12. Averaged UV-Vis spectra of NOM6 and NOL14.

Spectra calculated at ADC(2) / cc-pVDZ level from snapshots obtained using DFTB2 with dispersion correction. (A-F) The experimental mean absorption maximum is shown as a black solid line. Selected clusters are as follows: NOM6, 11-*cis* RSB with protonated D234 (λmax = 473 nm, ClusterBB 1 in (B); NOM6, all-*trans* RSB with deprotonated D234 (λmax = 566 nm, ClusterBB 2 in (C)); NOL14, 11-*cis* RSB (λmax = 594 nm, ClusterRBP 1 in (E)); and NOL14, all-*trans* RSB (λmax = 497 nm, ClusterRBP 3 in (F)). Note that NOM6 with 11-*cis* RSB and deprotonated D234 (A) and NOM6 with all-*trans* RSB and protonated D234 (D) were not selected, as their calculated spectral trends were inconsistent with the experimental observations.

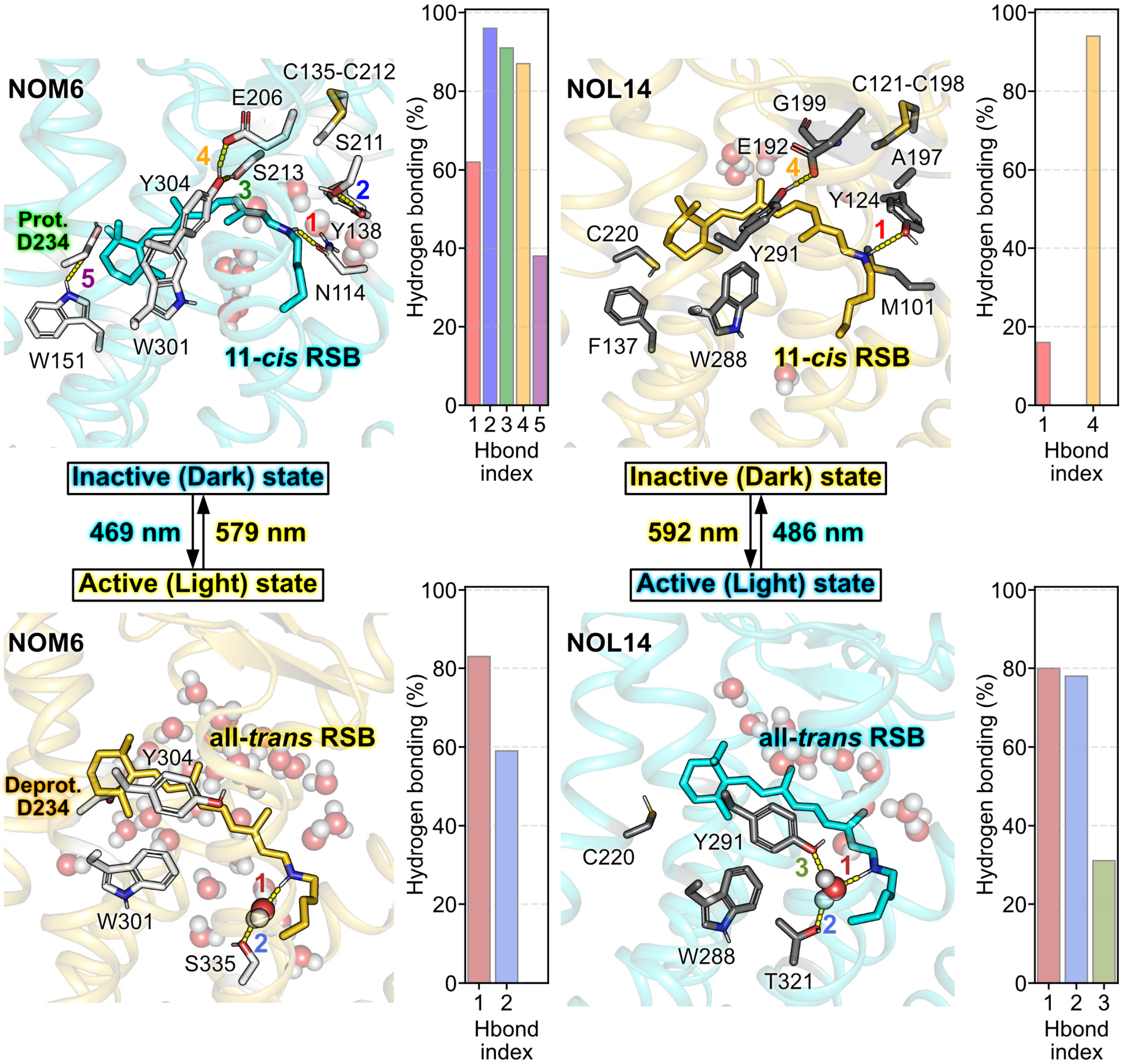

Fig. S13. Hydrogen bonding analysis of NOM6 and NOL14.

Percentage of hydrogen-bonding interactions were analyzed over 1-ns hybrid quantum mechanics / molecular mechanics (QM/MM) simulations using a distance cutoff of 3.5 Å between donor (D) and acceptor (A) atoms and an angle cutoff of 150° for the D-H-A geometry. Water molecules are shown as spheres.

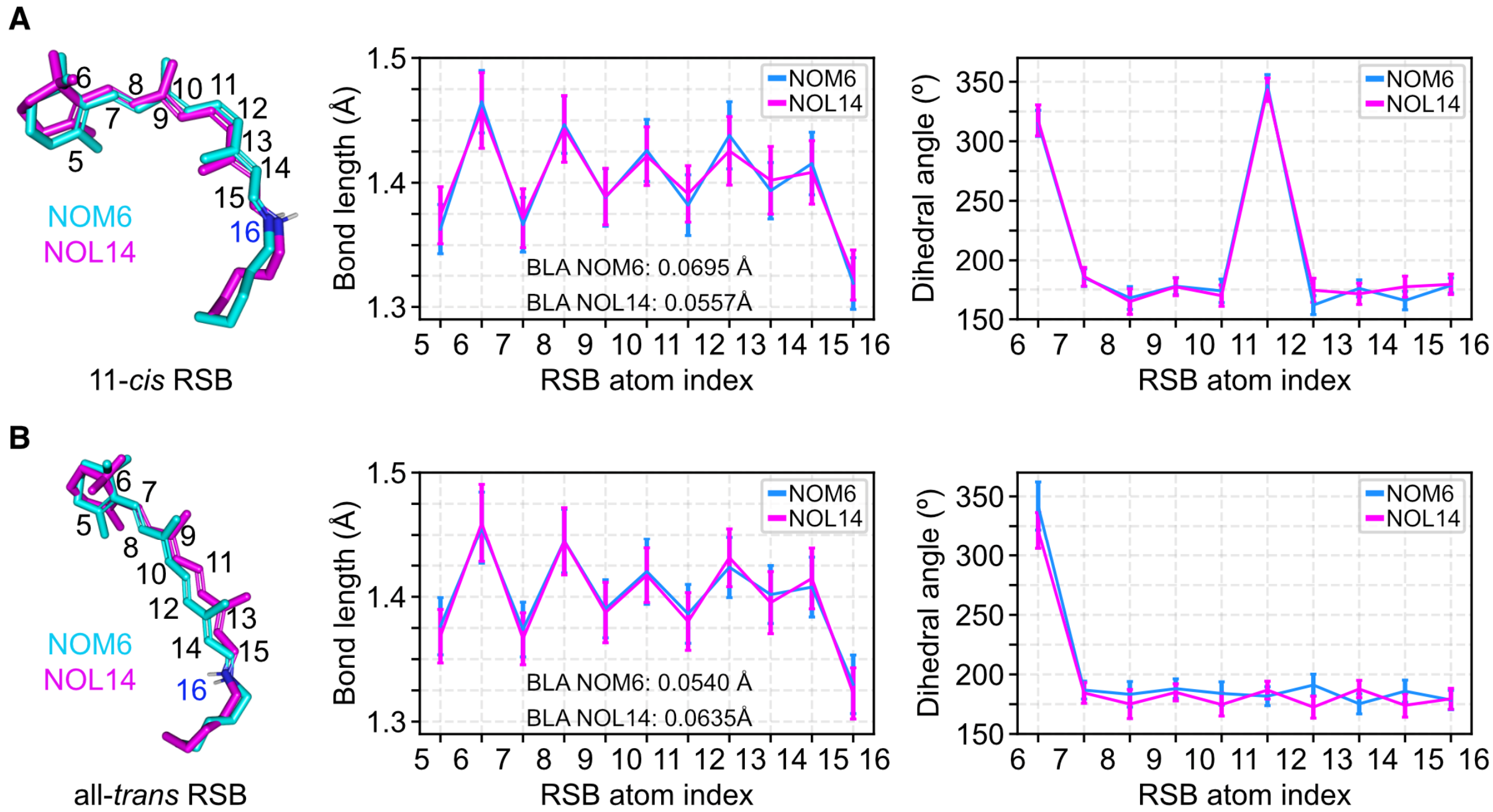

Fig. S14. Bond length and dihedral angle analysis.

Bond lengths and dihedral angles of (A) 11-*cis* and (B) all-*trans* retinal Schiff base bound in NOM6 (cyan) and NOL14 (magenta) were obtained from 1-ns hybrid quantum mechanics / molecular mechanics (QM/MM) simulations. The bond length alternation (BLA) was calculated as the difference between the average lengths of single and double bonds.

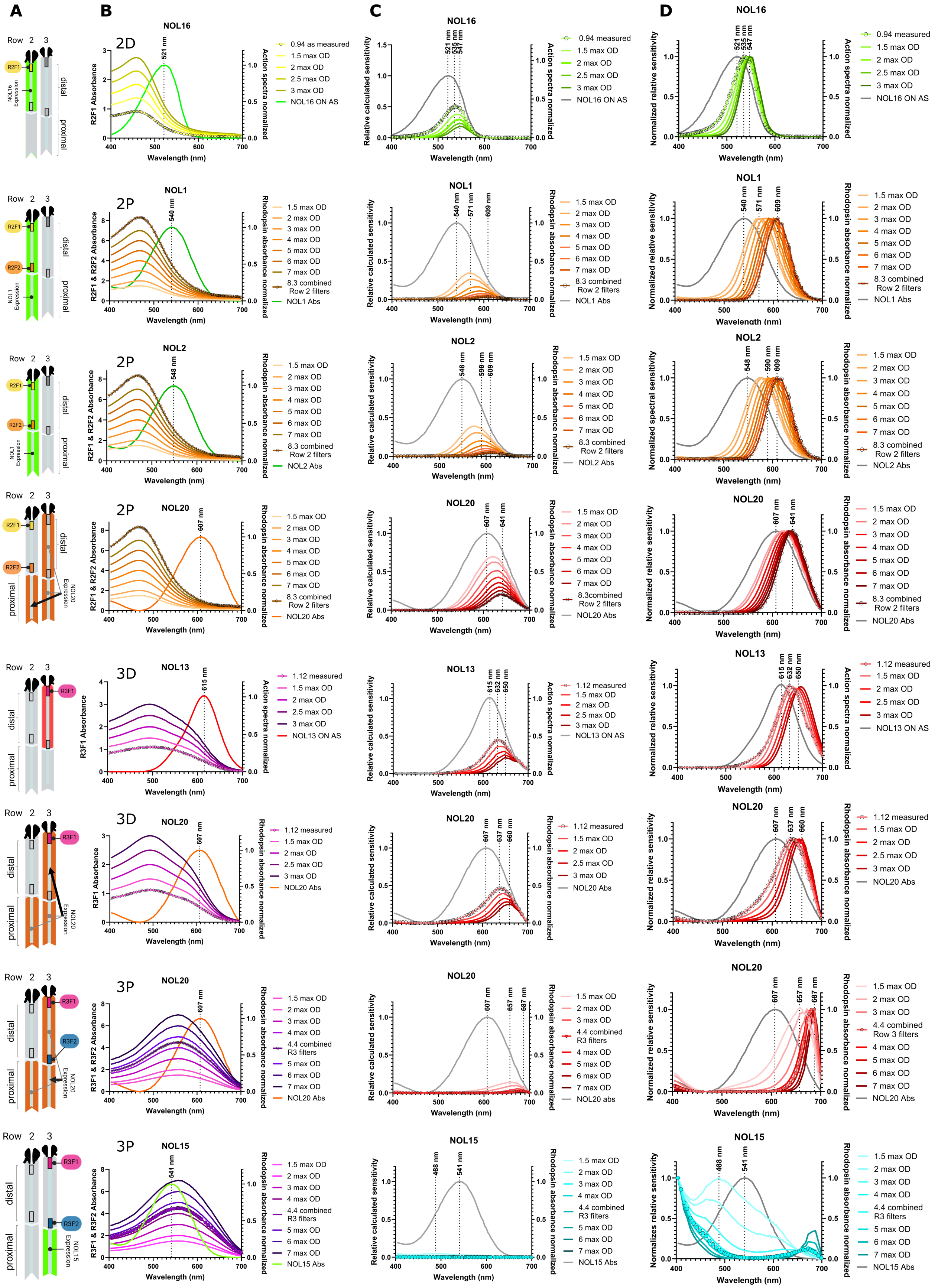
A
B
C
D

Fig. S15. Modeling the effect of intra-rhabdomal filter properties on the relative spectral sensitivity in tiers 2D, 2P, 3D and 3P.

(A) Schematic representation of midband rows 2 and 3 including the rhodopsins and the filters relevant for the analysis presented in each row. (B) Normalized rhodopsin spectra and filter spectra of different optical density ranging from 1.5 to 3 for distal (D) filters and ranging from 1.5 to 8.3 for combined filters in the proximal (P) tier. Digitized filter absorbances reported in the literature (*5*) are labelled by black circle symbols. (C) Calculated sensitivities of the indicated rhodopsins unfiltered (grey) and after filtering by screening pigments with maximal absorbance ranging from 1.5 to 3 for distal F1 filters and ranging from 1.5 to 8.3 for proximal F2 filters. Numbers above vertical dashed/dotted lines represent, from left to right, the wavelength maxima before screening, after screening by filters of reported optical densities (OD), and after maximal screening when applicable (NOL15 is almost entirely shielded by intra-rhabdomal pigments and the low resulting sensitivity could not be fitted). (D) Normalized calculated sensitivity spectra plotted with original rhodopsin spectrum (grey). In all graphs, absorbances and sensitivities resulting from originally measured filters (*5*) are labeled by black open circle symbols.

Table S1. Gq- and Gi/o-coupled response characteristics measured in the aequorin and glosensor assay.

Significant Gq and Gi/o-responses are reported in bold. (A). Maximal relative Gq-responses are expressed as a percentage of the highest Gq-signaling opsin (NOL19). (B). Maximal relative Gi/o responses are reported for each opsin, as the percentage of response of cells transfected only with glosensor (Glosensor only), at the peak decrease in luminescence, between the forskolin-induced plateau and the estimated response decay (see Materials and Methods).

		**Gq**				
Clade	*Opsin*	*Average rel. Luminescence*	*SEM*	*p-value*	*n*	*% of maximal response*
A	**NOL6**	**10.96**	**3.72**	**0.0296**	**5**	**2.5%**
	**NOL7**	**12.38**	**3.76**	**0.0207**	**5**	**2.8%**
	**NOL12**	**26.00**	**6.82**	**0.0338**	**3**	**5.8%**
	NOL8	1.33	0.13	0.3066	5	0.3%
-	**LWS R3**	**26.79**	**8.34**	**0.0274**	**4**	**6.0%**
C	NOL9	1.22	0.06	0.4211	4	0.3%
	NOL20	1.57	0.14	0.0328	5	0.4%
D	**NOL1**	**4.29**	**1.20**	**0.0424**	**4**	**1.0%**
	NOL2	1.27	0.10	0.3958	4	0.3%
E	**NOL16**	**357.30**	**65.89**	**0.0163**	**3**	**80.0%**
	**NOL17**	**127.60**	**28.86**	**0.0241**	**3**	**28.6%**
	**NOL19**	**446.60**	**65.57**	**0.0105**	**3**	**100.0%**
F	**NOL13**	**33.51**	**4.04**	**0.0021**	**4**	**7.5%**
	NOL15	1.41	0.17	0.3929	5	0.3%
	**NOL14**	**97.60**	**23.90**	**0.0078**	**5**	**21.9%**
I	**NOM5**	**342.30**	**34.43**	**0.0179**	**5**	**76.6%**
	**NOM6**	**252.50**	**42.94**	**0.0179**	**5**	**56.5%**

		**Gi/o**				
**Clade**	**Opsin**	**Average rel. luminescence**	**SEM**	**p-value**	**n**	**% of Glosensor only**
**A**	**NOL6**	**0.74**	**0.01**	**0.0278**	**3**	**114.8%**
	**NOL7**	**0.70**	**0.03**	**0.2**	**3**	**109.5%**
	**NOL12**	**0.78**	**0.02**	**0.0153**	**3**	**120.7%**
	**NOL8**	**0.63**	**0.02**	**0.3803**	**3**	**98.8%**
**-**	**LWS R3**	**0.66**	**0.00**	**0.3542**	**3**	**102.8%**

C	NOL9	0.73	0.03	0.0805	3	113.2%
	NOL20	0.94	0.03	0.0645	3	92.9%
D	NOL1	0.70	0.01	0.0961	3	108.7%
	NOL2	0.70	0.02	0.1273	3	108.7%
E	NOL16	0.72	0.02	0.0701	3	112.0%
	NOL17	0.75	0.02	0.0289	3	116.1%
	NOL19	0.49	0.02	0.0081	3	76.8%
F	NOL13	0.73	0.01	0.0318	3	114.2%
	NOL15	0.73	0.05	0.1051	3	113.6%
	NOL14	1.00	0.01	0.3224	6	104.4%
I	NOM5	0.73	0.01	<0.0001	3	73.8%
	NOM6	0.79	0.02	0.0006	3	79.2%

Table S2.

Dark state spectral characteristics of photoreversible opsins

The percentage of mixture for the individual gaussian is reported as the percentage of the area under the curve of the combined GMM model. See in addition fig. S8.

	Mixture	Gaussian 1		Gaussian 2		Light Intensity *(photons cm^{-2} s^{-1})*	*R^2*	*n*
	λ_{max} (nm)	*λ_{max} (nm)*	*% of mix*	*λ_{max} (nm)*	*% of mix*			
**NOL13**	615 ± 5.6	618.9	55.9	572.6	44.1	$1x10^{15}$	0.98	3
**NOL14**	592.4 ± 3.4	538.2	60.9	597.5	39.1	$1x10^{15}$	0.98	3
**NOM5**	474.9 ± 8.2	449.6	65.3	487.3	34.7	$2.4x10^{13}$	0.96	3
**NOM6**	469.1 ± 9.8	480.9	52.7	442.1	47.3	$2.4x10^{13}$	0.97	4

Table S3.

System setup for molecular dynamics simulations. ConfRSB: configuration of retinal Schiff base, Dim: box size (X/Y/Z nm), NNa/Cl: the number of Na+/Cl-, Nwater: the number of water molecules, NPOPC: the number of POPC. All simulations were conducted at a NaCl concentration of 150 mM and at a temperature of 303 K and replicated three times.

	$Conf_{RSB}$	*Dim*	$N_{Na/Cl}$	N_{water}	N_{POPC}
NOM6	11-*cis*	9.7/7.7/13.0	75/78	27929	260
(Prot. D234)	all-*trans*	9.5/9.5/14.4	83/86	30744	258
NOM6	11-*cis*	9.6/9.6/13.1	75/77	27935	260
(Deprot. D234)	all-*trans*	9.6/9.6/13.9	83/85	30740	258
NOL14	11-*cis*	7.9/7.9/14.4	57/61	21396	173
	all-*trans*	8.0/8.0/13.5	53/58	19937	172

Table S4.

Step-wise release of restraint of applied positional and dihedral angular restraints for energy minimization and equilibration simulations.

Time step: Δt (fs), individual simulation time: t (ns), force constant (kJ/mol/nm) for position/angle harmonic restraints of backbone heavy atoms (kbb), side-chain heavy atoms (ksc), side-chain heavy atoms of retinal Schiff base (kscRSB), lipid head group (khead), and lipid chirality and cis double bond (ktorsion).

	Δt	t	k_{bb}	k_{sc}	k_{scRSB}	k_{head}	$k_{torsion}$
Energy minimization	-	-	4000	2000	2000	1000	1000
Equilibration 1	1	0.25	4000	2000	2000	1000	1000
Equilibration 2	1	0.25	2000	1000	2000	400	400
Equilibration 3	1	0.25	1000	500	1000	400	200
Equilibration 4	2	1	500	200	1000	200	200
Equilibration 5	2	1	200	50	500	40	100
Equilibration 6	2	1	50	0	500	0	0
Equilibration 7	2	100	0	0	200	0	0

Table S5.

Dark state spectral characteristics of photoreversible opsins

The percentage of mixture for the individual gaussian is reported as the percentage of the area under the curve of the combined GMM model.

	RSB configuration	*BB 1*	*BB 2*	*BB 3*	*RBP 1*	*RBP 2*	*RBP 3*
NOM6	11-*cis*	95.1	2.8	1.8	72.2	27.1	0.4
(Prot. D234)	all-*trans*	76.9	12.6	9.8	98.5	0.8	0.7
NOM6	11-*cis*	73.6	15.0	7.5	61.1	22.6	10.1
(Deprot. D234)	all-*trans*	40.0	17.0	15.1	34.8	25.7	14.5
NOL14	11-*cis*	92.6	4.0	3.0	98.3	1.3	0.4
	all-*trans*	97.8	1.8	0.4	95.5	2.6	1.8

All generated data (Data S1 to S6) are available for download from the Dryad Dataset DOI: 10.5061/dryad.qz612jmxd.

Data S1. (.xlsx)
Contains all the sequences used for the alignments and phylogeny in this manuscript, including reference sequences and their accession numbers.

Data S2. (.xlsx)
Contains absorption spectra of dark adapted purified rhodopsins.

Data S3. (.xlsx)
Contains action spectra measurements.

Data S4. (.xlsx)
Contains G-coupling measurements with GsX and GqX to determine G-coupling specificity of rhodopsins.

Data S5. (.xlsx)
Contains G-coupling measurements with human Gs, Gi and Gq.

Data S6. (.xlsx)
Contains activity measurements for comparison of cloning strategies.

REFERENCES

1. T. W. Cronin, M. L. Porter, M. J. Bok, R. L. Caldwell, J. Marshall, Colour vision in stomatopod crustaceans. *Philos. Trans. R Soc. Lond. B Biol. Sci.* **377**, 20210278 (2022).

2. N. J. Marshall, M. F. Land, C. A. King, T. W. Cronin, The compound eyes of mantis shrimps (Crustacea, Hoplocarida, Stomatopoda). II. Colour pigments in the eyes of stomatopod crustaceans: Polychromatic vision by serial and lateral filtering. *Phil. Trans. R. Soc. B* **334**, 57–84 (1991).

3. J. Marshall, T. W. Cronin, S. Kleinlogel, Stomatopod eye structure and function: A review. *Arthropod Struct. Dev.* **36**, 420–448 (2007).

4. T. W. Cronin, N. J. Marshall, R. L. Caldwell, The intrarhabdomal filters in the retinas of mantis shrimps. *Vision Res.* **34**, 279–291 (1994).

5. T. W. Cronin, N. J. Marshall, Multiple spectral classes of photoreceptors in the retinas of gonodactyloid stomatopod crustaceans. *J. Comp. Physiol. A* **166**, 261–275 (1989).

6. T. Cronin, N. J. Marshall, R. L. Caldwell, N. Shashar, Specialization of retinal function in the compound eyes of mantis shrimps. *Vision Res.* **34**, 2639–2656 (1994).

7. J. Marshall, J. Oberwinkler, The colourful world of the mantis shrimp. *Nature* **401**, 873–874 (1999).

8. H. H. Thoen, N. J. Strausfeld, J. Marshall, Neural organization of afferent pathways from the stomatopod compound eye. *J. Comp. Neurol.* **525**, 3010–3030 (2017).

9. A. Streets, H. England, J. Marshall, Colour vision in stomatopod crustaceans: More questions than answers. *J. Exp. Biol.* **225**, jeb243699 (2022).

10. C.-W. J. Wang, N. J. Marshall, Behavioural evidence of spectral opponent processing in the visual system of stomatopod crustaceans. *J. Exp. Biol.* **228**, jeb247952 (2025).

11. T. W. Cronin, M. J. Bok, N. J. Marshall, R. L. Caldwell, Filtering and polychromatic vision in mantis shrimps: Themes in visible and ultraviolet vision. *Philos. Trans. R Soc. Lond. B Biol. Sci.* **369**, 20130032 (2014).

12. N. S. Roberts, J. F. D. Hagen, R. J. Johnston, The diversity of invertebrate visual opsins spanning Protostomia, Deuterostomia, and Cnidaria. *Dev. Biol.* **492**, 187–199 (2022).

13. T. W. Cronin, S. Johnsen, J. Marshall, E. J. Warrant, *Visual Ecology* (Princeton Univ. Press, 2014; https://press.princeton.edu/books/hardcover/9780691151847/visual-ecology).

14. M. A. Liénard, G. D. Bernard, A. Allen, J.-M. Lassance, S. Song, R. R. Childers, N. Yu, D. Ye, A. Stephenson, W. A. Valencia-Montoya, S. Salzman, M. R. L. Whitaker, M. Calonje, F. Zhang, N. E. Pierce, The evolution of red color vision is linked to coordinated rhodopsin tuning in lycaenid butterflies. *Proc. Natl. Acad. Sci. U.S.A.* **118**, e2008986118 (2021).

15. S. L. Merbs, J. Nathans, Absorption spectra of human cone pigments. *Nature* **356**, 433–435 (1992).

16. J. W. L. Parry, K. L. Carleton, T. Spady, A. Carboo, D. M. Hunt, J. K. Bowmaker, Mix and match color vision: Tuning spectral sensitivity by differential opsin gene expression in lake malawi cichlids. *Curr. Biol.* **15**, 1734–1739 (2005).

17. C. R. Sharkey, J. Blanco, N. P. Lord, T. J. Wardill, Jewel beetle opsin duplication and divergence is the mechanism for diverse spectral sensitivities. *Mol. Biol. Evol.* **40**, msad023 (2023).

18. T. C. Spady, J. W. L. Parry, P. R. Robinson, D. M. Hunt, J. K. Bowmaker, K. L. Carleton, Evolution of the cichlid visual palette through ontogenetic subfunctionalization of the opsin gene arrays. *Mol. Biol. Evol.* **23**, 1538–1547 (2006).

19. S. Yokoyama, Molecular evolution of vertebrate visual pigments. *Prog. Retin. Eye Res.* **19**, 385–419 (2000).

20. J. C. Corbo, Vitamin A1/A2 chromophore exchange: Its role in spectral tuning and visual plasticity. *Dev. Biol.* **475**, 145–155 (2021).

21. V. H. Corredor, E. Hauzman, A. D. S. Gonçalves, D. F. Ventura, Genetic characterization of the visual pigments of the red-eared turtle (*Trachemys scripta elegans*) and computational predictions of the spectral sensitivity. *J. Photochem. Photobiol.* **12**, 100141 (2022).

22. J. M. Enright, M. B. Toomey, S. Sato, S. E. Temple, J. R. Allen, R. Fujiwara, V. M. Kramlinger, L. D. Nagy, K. M. Johnson, Y. Xiao, M. J. How, S. L. Johnson, N. W. Roberts, V. J. Kefalov, F. P. Guengerich, J. C. Corbo, Cyp27c1 red-shifts the spectral sensitivity of photoreceptors by converting vitamin A1 into A2. *Curr. Biol.* **25**, 3048–3057 (2015).

23. D. Escobar-Camacho, M. E. R. Pierotti, V. Ferenc, D. M. T. Sharpe, E. Ramos, C. Martins, K. L. Carleton, Variable vision in variable environments: The visual system of an invasive cichlid (*Cichla monoculus*) in Lake Gatun, Panama. *J. Exp. Biol.* **222**, jeb188300 (2019).

24. E. R. Loew, V. I. Govardovskii, Photoreceptors and visual pigments in the red-eared turtle, *Trachemys scripta elegans*. *Vis. Neurosci.* **18**, 753–757 (2001).

25. J. C. Partridge, R. H. Douglas, Far-red sensitivity of dragon fish. *Nature* **375**, 21–22 (1995).

26. Y. Imamoto, Y. Shichida, Cone visual pigments. *Biochim. Biophys. Acta* **1837**, 664–673 (2014).

27. C. J. van der Kooi, D. G. Stavenga, K. Arikawa, G. Belušič, A. Kelber, Evolution of insect color vision: From spectral sensitivity to visual ecology. *Annu. Rev. Entomol.* **66**, 435–461 (2021).

28. M. L. Porter, H. Awata, M. J. Bok, T. W. Cronin, Exceptional diversity of opsin expression patterns in *Neogonodactylus oerstedii* (Stomatopoda) retinas. *Proc. Natl. Acad. Sci. U.S.A.* **117**, 8948–8957 (2020).

29. G. G. Kochendoerfer, S. W. Lin, T. P. Sakmar, R. A. Mathies, How color visual pigments are tuned. *Trends Biochem. Sci.* **24**, 300–305 (1999).

30. M. W. Donohue, K. L. Carleton, T. W. Cronin, Opsin expression in the central nervous system of the mantis shrimp *Neogonodactylus oerstedii*. *Biol. Bull.* **233**, 58–69 (2017).

31. I. G. Gleadall, T. Hariyama, Y. Tsukahara, The visual pigment chromophores in the retina of insect compound eyes, with special reference to the Coleoptera. *J. Insect Physiol.* **35**, 787–795 (1989).

32. T. H. Goldsmith, T. W. Cronin, The retinoids of seven species of mantis shrimp. *Vis. Neurosci.* **10**, 915–920 (1993).

33. T. Suzuki, E. Eguchi, A survey of 3-dehydroretinal as a visual pigment chromophore in various species of crayfish and other freshwater crustaceans. *Experientia* **43**, 1111–1113 (1987).

34. T. W. Cronin, N. J. Marshall, R. L. Caldwell, Visual pigment diversity in two genera of mantis shrimps implies rapid evolution (Crustacea; Stomatopoda). *J. Comp. Physiol. A* **179**, 371–384 (1996).

35. S. M. Marino, V. N. Gladyshev, Analysis and functional prediction of reactive cysteine residues. *J. Biol. Chem.* **287**, 4419–4425 (2012).

36. G. D. Bernard, Red-absorbing visual pigment of butterflies. *Science* **203**, 1125–1127 (1979).

37. H. J. Bailes, R. J. Lucas, Human melanopsin forms a pigment maximally sensitive to blue light ($\lambda max \approx 479$ nm) supporting activation of Gq/11 and Gi/o signalling cascades. *Proc. Biol. Sci.* **280**, 20122987 (2013).

38. A. Inoue, F. Raimondi, F. M. N. Kadji, G. Singh, T. Kishi, A. Uwamizu, Y. Ono, Y. Shinjo, S. Ishida, N. Arang, K. Kawakami, J. S. Gutkind, J. Aoki, R. B. Russell, Illuminating G-protein-coupling selectivity of GPCRs. *Cell* **177**, 1933–1947.e25 (2019).

39. E. R. Ballister, J. Rodgers, F. Martial, R. J. Lucas, A live cell assay of GPCR coupling allows identification of optogenetic tools for controlling Go and Gi signaling. *BMC Biol.* **16**, 10 (2018).

40. A. Inoue, J. Ishiguro, H. Kitamura, N. Arima, M. Okutani, A. Shuto, S. Higashiyama, T. Ohwada, H. Arai, K. Makide, J. Aoki, TGFα shedding assay: An accurate and versatile method for detecting GPCR activation. *Nat. Methods* **9**, 1021–1029 (2012).

41. R. J. McDowell, A. Didikoglu, T. Woelders, M. J. Gatt, F. Moffatt, S. Notash, R. A. Hut, T. M. Brown, R. J. Lucas, Beyond Lux: Methods for species and photoreceptor-specific quantification of ambient light for mammals. *BMC Biol.* **22**, 257 (2024).

42. K. W. Foster, "Chapter 3: Action spectroscopy of photomovement," in *Comprehensive Series in Photosciences*, D.-P. Häder, A. M. Breure, Eds. (Elsevier, 2001), vol. 1 of *Photomovement*, pp. 51–115. www.sciencedirect.com/science/article/pii/S1568461X01800077.

43. R. G. Foster, M. W. Hankins, Circadian vision. *Curr. Biol.* **17**, R746–R751 (2007).

44. H. Tsukamoto, A. Terakita, Diversity and functional properties of bistable pigments. *Photochem. Photobiol. Sci.* **9**, 1435–1443 (2010).

45. M. Koyanagi, B. Shen, T. Nagata, L. Sun, S. Wada, S. Kamimura, E. Kage-Nakadai, A. Terakita, High-performance optical control of GPCR signaling by bistable animal opsins MosOpn3 and LamPP in a molecular property–dependent manner. *Proc. Natl. Acad. Sci. U.S.A.* **119**, e2204341119 (2022).

46. A. Wagdi, D. Malan, U. Sathyanarayanan, J. S. Beauchamp, M. Vogt, D. Zipf, T. Beiert, B. Mansuroglu, V. Dusend, M. Meininghaus, L. Schneider, B. Kalthof, J. S. Wiegert, G. M. König, E. Kostenis, R. Patejdl, P. Sasse, T. Bruegmann, Selective optogenetic control of Gq signaling using human neuropsin. *Nat. Commun.* **13**, 1765 (2022).

47. H. J. Bailes, L.-Y. Zhuang, R. J. Lucas, Reproducible and sustained regulation of Gαs signalling using a metazoan opsin as an optogenetic tool. *PLOS ONE* **7**, e30774 (2012).

48. J. Abramson, J. Adler, J. Dunger, R. Evans, T. Green, A. Pritzel, O. Ronneberger, L. Willmore, A. J. Ballard, J. Bambrick, S. W. Bodenstein, D. A. Evans, C.-C. Hung, M. O'Neill, D. Reiman, K. Tunyasuvunakool, Z. Wu, A. Žemgulytė, E. Arvaniti, C. Beattie, O. Bertolli, A. Bridgland, A. Cherepanov, M. Congreve, A. I. Cowen-Rivers, A. Cowie, M. Figurnov, F. B. Fuchs, H. Gladman, R. Jain, Y. A. Khan, C. M. R. Low, K. Perlin, A. Potapenko, P. Savy, S. Singh, A. Stecula, A. Thillaisundaram, C. Tong, S. Yakneen, E. D. Zhong, M. Zielinski, A. Žídek, V. Bapst, P. Kohli, M. Jaderberg, D. Hassabis, J. M. Jumper,

Accurate structure prediction of biomolecular interactions with AlphaFold 3. *Nature* **630**, 493–500 (2024).

49. R. A. Stein, H. S. Mchaourab, SPEACH_AF: Sampling protein ensembles and conformational heterogeneity with Alphafold2. *PLOS Comput. Biol.* **18**, e1010483 (2022).

50. R. Sato, A. Terakita, M. Koyanagi, Dragonfly red opsins share a common tuning mechanism with mammalian red opsins and further enhancement of near-infrared sensitivity. *Cell. Mol. Life Sci.* **83**, 66 (2026).

51. B. Honig, U. Dinur, K. Nakanishi, V. Balogh-Nair, M. A. Gawinowicz, M. Arnaboldi, M. G. Motto, An external point-charge model for wavelength regulation in visual pigments. *J. Am. Chem. Soc.* **101**, 7084–7086 (1979).

52. K. Nakanishi, V. Balogh-Nair, M. Arnaboldi, K. Tsujimoto, B. Honig, An external point-charge model for bacteriorhodopsin to account for its purple color. *J. Am. Chem. Soc.* **102**, 7945–7947 (1980).

53. E. J. Warrant, S. Johnsen, Vision and the light environment. *Curr. Biol.* **23**, R990–R994 (2013).

54. S. Kleinlogel, N. J. Marshall, Electrophysiological evidence for linear polarization sensitivity in the compound eyes of the stomatopod crustacean *Gonodactylus chiragra*. *J. Exp. Biol.* **209**, 4262–4272 (2006).

55. T. W. Cronin, N. J. Marshall, C. A. Quinn, C. A. King, Ultraviolet photoreception in mantis shrimp. *Vision Res.* **34**, 1443–1452 (1994).

56. T. D. Lamb, Photoreceptor spectral sensitivities: Common shape in the long-wavelength region. *Vision Res.* **35**, 3083–3091 (1995).

57. J. H. Dominguez, M. L. Reaka, Temporal activity patterns in reef-dwelling stomatopods: A test of alternative hypotheses. *J. Exp. Mar. Biol. Ecol.* **117**, 47–69 (1988).

58. T.-H. Chiou, N. J. Marshall, R. L. Caldwell, T. W. Cronin, Changes in light-reflecting properties of signalling appendages alter mate choice behaviour in a stomatopod crustacean *Haptosquilla trispinosa*. *Mar. Freshw. Behav. Physiol.* **44**, 1–11 (2011).

59. A. M. Franklin, M. B. Applegate, S. M. Lewis, F. G. Omenetto, Stomatopods detect and assess achromatic cues in contests. *Behav. Ecol.* **28**, 1329–1336 (2017).

60. A. M. Franklin, J. Marshall, A. D. Feinstein, M. J. Bok, A. D. Byrd, S. M. Lewis, Differences in signal contrast and camouflage among different colour variations of a stomatopod crustacean, *Neogonodactylus oerstedii*. *Sci. Rep.* **10**, 1236 (2020).

61. M. Koyanagi, S. Wada, E. Kawano-Yamashita, Y. Hara, S. Kuraku, S. Kosaka, K. Kawakami, S. Tamotsu, H. Tsukamoto, Y. Shichida, A. Terakita, Diversification of non-visual photopigment parapinopsin in spectral sensitivity for diverse pineal functions. *BMC Biol.* **13**, 73 (2015).

62. J. Wietek, A. Nozownik, M. Pulin, I. Saraf-Sinik, N. Matosevich, R. Gowrishankar, A. Gat, D. Malan, B. J. Brown, J. Dine, B. N. Imambocus, R. Levy, K. Sauter, A. Litvin, N. Regev, S. Subramaniam, K. Abrera, D. Summarli, E. M. Goren, G. Mizrachi, E. Bitton, A. Benjamin, B. A. Copits, P. Sasse, B. R. Rost, D. Schmitz, M. R. Bruchas, P. Soba, M. Oren-Suissa, Y. Nir, J. S. Wiegert, O. Yizhar, A bistable inhibitory optoGPCR for multiplexed optogenetic control of neural circuits. *Nat. Methods* **21**, 1275–1287 (2024).

63. T. Nagata, M. Koyanagi, H. Tsukamoto, E. Mutt, G. F. X. Schertler, X. Deupi, A. Terakita, The counterion–retinylidene Schiff base interaction of an invertebrate rhodopsin rearranges upon light activation. *Commun. Biol.* **2**, 180 (2019).

64. M. J. Rodrigues, O. Tejero, J. Mühle, F. Pamula, I. Das, C.-J. Tsai, A. Terakita, M. Sheves, G. F. X. Schertler, Activating an invertebrate bistable opsin with the all-trans 6.11 retinal analog. *Proc. Natl. Acad. Sci. U.S.A.* **121**, e2406814121 (2024).

65. A. Terakita, R. Hara, T. Hara, Retinal-binding protein as a shuttle for retinal in the rhodopsin-retinochrome system of the squid visual cells. *Vision Res.* **29**, 639–652 (1989).

66. K. Inoue, M. Tsuda, M. Terazima, Photoreverse reaction dynamics of octopus rhodopsin. *Biophys. J.* **92**, 3643–3651 (2007).

67. M. A. Larkin, G. Blackshields, N. P. Brown, R. Chenna, P. A. McGettigan, H. McWilliam, F. Valentin, I. M. Wallace, A. Wilm, R. Lopez, J. D. Thompson, T. J. Gibson, D. G. Higgins, Clustal W and Clustal X version 2.0. *Bioinformatics* **23**, 2947–2948 (2007).

68. D. G. Gibson, L. Young, R.-Y. Chuang, J. C. Venter, C. A. Hutchison, H. O. Smith, Enzymatic assembly of DNA molecules up to several hundred kilobases. *Nat. Methods* **6**, 343–345 (2009).

69. C.-F. Bowin, A. Inoue, G. Schulte, WNT-3A–induced β-catenin signaling does not require signaling through heterotrimeric G proteins. *J. Biol. Chem.* **294**, 11677–11684 (2019).

70. V. I. Govardovskii, N. Fyhrquist, T. Reuter, D. G. Kuzmin, K. Donner, In search of the visual pigment template. *Vis. Neurosci.* **17**, 509–528 (2000).

71. P. Virtanen, R. Gommers, T. E. Oliphant, M. Haberland, T. Reddy, D. Cournapeau, E. Burovski, P. Peterson, W. Weckesser, J. Bright, S. J. van der Walt, M. Brett, J. Wilson, K. J. Millman, N. Mayorov, A. R. J. Nelson, E. Jones, R. Kern, E. Larson, C. J. Carey, İ. Polat, Y. Feng, E. W. Moore, J. VanderPlas, D. Laxalde, J. Perktold, R. Cimrman, I. Henriksen, E. A. Quintero, C. R. Harris, A. M. Archibald, A. H. Ribeiro, F. Pedregosa, P. van Mulbregt, SciPy 1.0: Fundamental algorithms for scientific computing in Python. *Nat. Methods* **17**, 261–272 (2020).

72. F. Pedregosa, G. Varoquaux, A. Gramfort, V. Michel, B. Thirion, O. Grisel, M. Blondel, A. Müller, J. Nothman, G. Louppe, P. Prettenhofer, R. Weiss, V. Dubourg, J. Vanderplas, A. Passos, D. Cournapeau, M. Brucher, M. Perrot, É. Duchesnay, Scikit-learn: Machine learning in Python. arXiv:1201.0490 [cs.LG] (2018).

73. L. S. Brown, Fungal rhodopsins and opsin-related proteins: Eukaryotic homologues of bacteriorhodopsin with unknown functions. *Photochem. Photobiol. Sci.* **3**, 555–565 (2004).

74. J. Schindelin, I. Arganda-Carreras, E. Frise, V. Kaynig, M. Longair, T. Pietzsch, S. Preibisch, C. Rueden, S. Saalfeld, B. Schmid, J.-Y. Tinevez, D. J. White, V. Hartenstein, K. Eliceiri, P. Tomancak, A. Cardona, Fiji: An open-source platform for biological-image analysis. *Nat. Methods* **9**, 676–682 (2012).

75. M. Mirdita, K. Schütze, Y. Moriwaki, L. Heo, S. Ovchinnikov, M. Steinegger, ColabFold: Making protein folding accessible to all. *Nat. Methods* **19**, 679–682 (2022).

76. C. J. Williams, J. J. Headd, N. W. Moriarty, M. G. Prisant, L. L. Videau, L. N. Deis, V. Verma, D. A. Keedy, B. J. Hintze, V. B. Chen, S. Jain, S. M. Lewis, W. B. Arendall III, J. Snoeyink, P. D. Adams, S. C. Lovell, J. S. Richardson, D. C. Richardson, MolProbity: More and better reference data for improved all-atom structure validation. *Protein Sci.* **27**, 293–315 (2018).

77. L. S. Johnson, S. R. Eddy, E. Portugaly, Hidden Markov model speed heuristic and iterative HMM search procedure. *BMC Bioinformatics* **11**, 431 (2010).

78. M. Steinegger, J. Söding, MMseqs2 enables sensitive protein sequence searching for the analysis of massive data sets. *Nat. Biotechnol.* **35**, 1026–1028 (2017).

79. D. Liebschner, P. V. Afonine, M. L. Baker, G. Bunkóczi, V. B. Chen, T. I. Croll, B. Hintze, L.-W. Hung, S. Jain, A. J. McCoy, N. W. Moriarty, R. D. Oeffner, B. K. Poon, M. G. Prisant, R. J. Read, J. S. Richardson, D. C. Richardson, M. D. Sammito, O. V. Sobolev, D. H. Stockwell, T. C. Terwilliger, A. G. Urzhumtsev, L. L. Videau, C. J. Williams, P. D. Adams, Macromolecular structure determination using X-rays, neutrons and electrons: Recent developments in Phenix. *Acta Crystallogr. D Struct. Biol.* **75**, 861–877 (2019).

80. A. Šali, T. L. Blundell, Comparative protein modelling by satisfaction of spatial restraints. *J. Mol. Biol.* **234**, 779–815 (1993).

81. G. Jones, P. Willett, R. C. Glen, A. R. Leach, R. Taylor, Development and validation of a genetic algorithm for flexible docking1. *J. Mol. Biol.* **267**, 727–748 (1997).

82. M. Murakami, T. Kouyama, Crystal structure of squid rhodopsin. *Nature* **453**, 363–367 (2008).

83. T. Gruhl, T. Weinert, M. J. Rodrigues, C. J. Milne, G. Ortolani, K. Nass, E. Nango, S. Sen, P. J. M. Johnson, C. Cirelli, A. Furrer, S. Mous, P. Skopintsev, D. James, F. Dworkowski, P. Båth, D. Kekilli, D. Ozerov, R. Tanaka, H. Glover, C. Bacellar, S. Brünle, C. M. Casadei, A. D. Diethelm, D. Gashi, G. Gotthard, R. Guixà-González, Y. Joti, V. Kabanova, G. Knopp, E. Lesca, P. Ma, I. Martiel, J. Mühle, S. Owada, F. Pamula, D. Sarabi, O. Tejero, C.-J. Tsai, N. Varma, A. Wach, S. Boutet, K. Tono, P. Nogly, X. Deupi, S. Iwata, R. Neutze, J. Standfuss, G. Schertler, V. Panneels, Ultrafast structural changes direct the first molecular events of vision. *Nature* **615**, 939–944 (2023).

84. S. Zhang, J. M. Krieger, Y. Zhang, C. Kaya, B. Kaynak, K. Mikulska-Ruminska, P. Doruker, H. Li, I. Bahar, ProDy 2.0: Increased scale and scope after 10 years of protein dynamics modelling with Python. *Bioinformatics* **37**, 3657–3659 (2021).

85. M. J. Abraham, T. Murtola, R. Schulz, S. Páll, J. C. Smith, B. Hess, E. Lindahl, GROMACS: High performance molecular simulations through multi-level parallelism from laptops to supercomputers. *SoftwareX* **1-2**, 19–25 (2015).

86. J. Huang, S. Rauscher, G. Nawrocki, T. Ran, M. Feig, B. L. de Groot, H. Grubmüller, A. D. MacKerell, CHARMM36m: An improved force field for folded and intrinsically disordered proteins. *Nat. Methods* **14**, 71–73 (2017).

87. W. L. Jorgensen, J. Chandrasekhar, J. D. Madura, R. W. Impey, M. L. Klein, Comparison of simple potential functions for simulating liquid water. *J. Chem. Phys.* **79**, 926–935 (1983).

88. S. Jo, T. Kim, V. G. Iyer, W. Im, CHARMM-GUI: A web-based graphical user interface for CHARMM. *J. Comput. Chem.* **29**, 1859–1865 (2008).

89. E. Tajkhorshid, B. Paizs, S. Suhai, Conformational effects on the proton affinity of the schiff base in bacteriorhodopsin: A density functional study. *J. Phys. Chem. B* **101**, 8021–8028 (1997).

90. E. Tajkhorshid, S. Suhai, Influence of the methyl groups on the structure, charge distribution, and proton affinity of the retinal schiff base. *J. Phys. Chem. B* **103**, 5581–5590 (1999).

91. E. Tajkhorshid, J. Baudry, K. Schulten, S. Suhai, Molecular dynamics study of the nature and origin of retinal's twisted structure in bacteriorhodopsin. *Biophys. J.* **78**, 683–693 (2000).

92. T. Darden, D. York, L. Pedersen, Particle mesh Ewald: An N·log(N) method for Ewald sums in large systems. *J. Chem. Phys.* **98**, 10089–10092 (1993).

93. G. Bussi, D. Donadio, M. Parrinello, Canonical sampling through velocity rescaling. *J. Chem. Phys.* **126**, 014101 (2007).

94. H. J. C. Berendsen, J. P. M. Postma, W. F. van Gunsteren, A. DiNola, J. R. Haak, Molecular dynamics with coupling to an external bath. *J. Chem. Phys.* **81**, 3684–3690 (1984).

95. M. Parrinello, Crystal structure and pair potentials: A molecular-dynamics study. *Phys. Rev. Lett.* **45**, 1196–1199 (1980).

96. X. Daura, K. Gademann, B. Jaun, D. Seebach, W. F. van Gunsteren, A. E. Mark, Peptide folding: When simulation meets experiment. *Angew. Chem. Int. Ed.* **38**, 236–240 (1999).

97. R. Salomon-Ferrer, D. A. Case, R. C. Walker, An overview of the Amber biomolecular simulation package. *WIREs Comput. Mol. Sci.* **3**, 198–210 (2013).

98. M. Elstner, Self-consistent-charge density-functional tight-binding method for simulations of complex materials properties. *Phys. Rev. B* **58**, 7260–7268 (1998).

99. M. Elstner, P. Hobza, T. Frauenheim, S. Suhai, E. Kaxiras, Hydrogen bonding and stacking interactions of nucleic acid base pairs: A density-functional-theory based treatment. *J. Chem. Phys.* **114**, 5149–5155 (2001).

100. J. Schirmer, Beyond the random-phase approximation: A new approximation scheme for the polarization propagator. *Phys. Rev. A* **26**, 2395–2416 (1982).

101. S. G. Balasubramani, G. P. Chen, S. Coriani, M. Diedenhofen, M. S. Frank, Y. J. Franzke, F. Furche, R. Grotjahn, M. E. Harding, C. Hättig, A. Hellweg, B. Helmich-Paris, C. Holzer, U. Huniar, M. Kaupp, A. Marefat Khah, S. Karbalaei Khani, T. Müller, F. Mack, B. D. Nguyen, S. M. Parker, E. Perlt, D. Rappoport, K. Reiter, S. Roy, M. Rückert, G. Schmitz, M. Sierka, E. Tapavicza, D. P. Tew, C. van Wüllen, V. K. Voora, F. Weigend, A. Wodyński, J. M. Yu, TURBOMOLE: Modular program suite for ab initio quantum-chemical and condensed-matter simulations. *J. Chem. Phys.* **152**, 184107 (2020).

102. K. Katoh, J. Rozewicki, K. D. Yamada, MAFFT online service: Multiple sequence alignment, interactive sequence choice and visualization. *Brief. Bioinform.* **20**, 1160–1166 (2019).

103. M. A. Liénard, W. A. Valencia-Montoya, N. E. Pierce, Molecular advances to study the function, evolution and spectral tuning of arthropod visual opsins. *Philos Trans. R Soc. Lond. B Biol. Sci.* **377**, 20210279 (2022).

104. Q. Zhou, D. Yang, M. Wu, Y. Guo, W. Guo, L. Zhong, X. Cai, A. Dai, W. Jang, E. I. Shakhnovich, Z.-J. Liu, R. C. Stevens, N. A. Lambert, M. M. Babu, M.-W. Wang, S. Zhao, Common activation mechanism of class A GPCRs. *eLife* **8**, e50279 (2019).
